# Phenotypic characterization and gene mapping of hybrid necrosis in *Triticum durum–Haynaldia villosa* amphiploids

**DOI:** 10.1007/s00122-024-04691-0

**Published:** 2024-07-15

**Authors:** Yangqi Liu, Jinhong Liu, Zhenpu Huang, Kaiwen Fan, Xinshuo Guo, Liping Xing, Aizhong Cao

**Affiliations:** 1 National Key Laboratory of Crop Genetics and Germplasm Enhancement, Nanjing Agricultural University/JCIC-MCP, Nanjing, 210095 China; 2 Zhongshan Biological Breeding Laboratory, Nanjing, 210014 China

## Abstract

**Key message:**

Phenotypical, physiological and genetic characterization was carried out on the hybrid necrosis gene from *Haynaldia villosa*, and the related gene *Ne-V* was mapped to chromosome arm 2VL.

**Abstract:**

Introducing genetic variation from wild relatives into common wheat through wide crosses is a vital strategy for enriching genetic diversity and promoting wheat breeding. However, hybrid necrosis, a genetic autoimmunity syndrome, often occurs in the offspring of interspecific or intraspecific crosses, restricting both the selection of hybrid parents and the pyramiding of beneficial genes. To utilize the germplasms of *Haynaldia villosa* (2*n* = 2*x* = 14, VV), we conducted wide hybridization between durum wheat (2*n* = 4*x* = 28, AABB) and multiple *H. villosa* accessions to synthesize the amphiploids (2*n* = 6*x* = 42, AABBVV). This study revealed that 61.5% of amphiploids derived from the above crosses exhibited hybrid necrosis, with some amphiploids even dying before reaching maturity. However, the initiation time and severity of necrosis varied dramatically among the progenies, suggesting that there were multiple genetic loci or multiple alleles in the same genetic locus conferring to hybrid necrosis in *H. villosa* accessions. Genetic analysis was performed on the F_2_ and derived F_2:3_ populations, which were constructed between amphiploid STH59-1 with normal leaves and amphiploid STH59-2 with necrotic leaves. A semidominant hybrid necrosis-related gene, *Ne-V,* was mapped to an 11.8-cM genetic interval on the long arm of chromosome 2V, representing a novel genetic locus identified in *Triticum-*related species. In addition, the hybrid necrosis was correlated with enhanced H_2_O_2_ accumulation and cell death, and it was influenced by the temperature and light. Our findings provide a foundation for cloning the *Ne-V* gene and exploring its molecular mechanism.

**Supplementary Information:**

The online version contains supplementary material available at 10.1007/s00122-024-04691-0.

## Introduction

Hybrid necrosis in plants refers to a genetic autoimmune syndrome occurring in the first or subsequent generations of interspecific or intraspecific hybrid plants (Li et al. 2021). It is favorable for reproductive isolation but harmful to gene flow among species (Chen et al. 2016). Weak hybrid necrosis typically leads to leaf necrosis and poor growth, whereas severe hybrid necrosis can result in premature death of leaves and even the entire plant (Hermsen 1963; Zhang et al. 2022). The Dobzhansky–Muller model provides a widely accepted mechanism for the evolution of reproductive isolation: incompatible substitutions disrupt interactions between genes (Bomblies and Weigel 2007; Morgan et al. 2020). The genetics of hybrid necrosis involve deleterious epistatic interactions between alleles at two or more loci, resulting in reduced fitness in the hybrids but not in their parents (Seehausen et al. 2014). The isolated gene pairs that contribute to hybrid necrosis have been intensively studied in various plant species, such as *Hwi1* and *Hwi2* (Chen et al. 2014), *hbd2* and *hbd3* (Yamamoto et al. 2010) in rice, *DM2d* and *DM1* (Bomblies et al. 2007), *DM2h* and *DM3* (Chae et al. 2014), *DM6* and *DM7* (Barragan et al. 2019), *DM8 (RPP4/5)* (Chae et al. 2014)*, DM9* (*ACD6*) (Świadek et al. 2017), *DM2h (RPP1)* and *SRF3* (Alcázar et al. 2010; Ordon et al. 2021), *DM11* and *DM10* (Barragan et al. 2021) in *Arabidopsis*, *Cf-2* and *Rcr3* in tomato (Krüger et al. 2002), and *RIN4* and *Dm39* in lettuce (Jeuken et al. 2009).

In common wheat (*Triticum aestivum* L., 2*n* = 6*x* = 42, AABBDD), hybrid necrosis was initially reported by Caldwell and Compton (1943). It has become a common occurrence in breeding panels during intraspecific and interspecific hybridization between various *Triticeae* species in recent decades. Hybrid weakness of wheat can be divided into hybrid necrosis, hybrid chlorosis and hybrid dwarfing.

Hybrid necrosis can be divided into four types (Type I through Type IV) based on its source and symptoms (Takamatsu et al. 2015). A pair of dominant loci, *Ne1* and *Ne2* on chromosome arms 5BL and 2BS, respectively, are responsible for Type I hybrid necrosis. Both the *Ne1* and *Ne2* loci carry multiple alleles, and different combinations of alleles can lead to different degrees of necrosis symptoms (Hermsen 1963; Chu et al. 2006; Vikas et al. 2013). The *Ne1* and *Ne2* have been precisely mapped, and *Ne2* encodes a coiled-coil nucleotide-binding site leucine-rich repeat (NLR) protein (Chu et al. 2006; Hewitt et al. 2021; Li et al. 2021; Si et al. 2021a, 2021b; Yan et al. 2021). Type II hybrid necrosis in wheat is controlled by the interaction of *Net1* from the AB genome of durum wheat and *Net2* from chromosome arm 2DS of *Aegilops tauschii* Cosson (2*n* = 2*x* = 14, DD). A genetic assay indicated that *Net2* was a semidominant gene, with *Net2Net2* plants showing severe necrosis, while *Net2net2* plants showing moderate necrosis (Mizuno et al. 2011; Sakaguchi et al. 2016). Type III hybrid necrosis is induced by the interaction of *Nec1* from chromosome arm 7DS and *Nec2* from durum wheat, and cell death is initiated in older tissues (Takamatsu et al. 2015; Nakano et al. 2015). Type IV hybrid necrosis occurs in interspecific hybrids of two wild diploid *Triticum* species, such as *T. monococcum* subsp. *aegilopoides* (Link) Thell. (2*n* = 2*x* = 14, A^m^A^m^) and *T. urartu* Tumanian ex Gandilyan (2*n* = 2*x* = 14, AA) (Yamagishi 1987; Takamatsu et al. 2015).

Three types of hybrid chlorosis (Type I, Type II and Type III) have been reported. Type I hybrid chlorosis with lethality is caused by the interaction between *Ch1* on chromosome arm 2A and *Ch2* on chromosome arm 3DL in tetraploid and hexaploid wheat (Mori and Tsunewaki 1992). For Type II hybrid chlorosis, two dominant complementary genes, *Cs1* on chromosome arm 5A and *Cs2* on chromosome arm 4G, were detected between *T. turgidum* subsp. *dicoccon* (Schrank) Thell. (2*n* = 4*x* = 28, AABB) and *T. timopheevii* Zhuk. (2*n* = 4*x* = 28, AAGG) (Tsunewaki 1992). Type III hybrid chlorosis was found in ABD triploids derived from interspecific crosses between tetraploid wheat and *Ae. tauschii*, and it was controlled by the single-gene *Hch1* on chromosome arm 7DS of *Ae. tauschii* (Nakano et al. 2015).

Hybrid dwarfing, or grass-clump dwarfness, is induced by the interaction of three dominant genes, namely *D1*, *D2* and *D3* (Ben Amer et al. 1996). In interspecific hybrids between wheat and rye (*Secale cereale* L.), a single allelic gene designated as *Hdw-R1* (*Hybrid dwarf-R1*) responsible for the dwarf phenotype was found from rye lines (Tikhenko et al. 2015). Moreover, some hybrid necrosis-related genes, such as *Ner1* and *Ner2*, have been identified in the R genome of rye (Ren and Lelley 1989).

The phenotype of hybrid necrosis is similar to that of immune responses (Kostoff 1930). Cloning of many genes for disease resistance and hybrid necrosis over the past few years revealed that a majority of hybrid necrosis genes were NLR-type genes, such as *DM1*, *DM2*, *DM4*, *DM5*, *DM10*, *RPP1* and *RPP7* in *Arabidopsis*, *Le4* in *Gossypium* and *hbd3* in rice, which represents the primary type of disease resistance gene (Chae et al. 2014; Barragan et al. 2021; Alcázar et al. 2014; Barragan et al. 2019; Deng et al. 2019; Yamamoto et al. 2010). In wheat, *Ne2*^*m*^*/els1* is the same gene as *LrZH22/Lr13* that encodes an NLR protein that confers resistance to leaf rust. The hypersensitive responses mediated by *Ne2* are responsive to activating both hybrid necrosis and disease resistance (Yan et al. 2021; Hewitt et al. 2021). In some cases, the autoimmune reaction of hybrid necrosis can also be triggered by receptor-like proteins (RLPs), like *Cf-9*, or receptor-like kinases (RLKs), such as *Hwi1* (Krüger et al. 2002; Chen et al. 2014).

*Haynadia villosa* (L.) Schur (2*n* = 2*x* = 14, VV) is an annual diploid species that originated in the northeast Mediterranean and southeast Europe. It shows high genetic diversity and harbors many desirable traits for improving wheat, such as high tiller number, tolerance to cold, drought and barren land, high grain protein content and resistance to powdery mildew, rust and yellow mosaic virus diseases (Zhang et al. 2014, 2016, 2021, 2005; Qi et al. 2011; Li et al. 2019; Dai et al. 2020). Therefore, transferring elite genes from *H. villosa* to common wheat through wide hybridization is a crucial approach for enhancing genetic diversity and promoting wheat improvement. We performed interspecific crosses between tetraploid durum wheat (*T. durum* Desf., 2*n* = 4*x* = 28, AABB) and multiple diploid *H. villosa* accessions and then conducted artificial allopolyploidization to create synthetic hexaploid amphiploids with an AABBVV genome. However, hybrid sterility, hybrid lethality, hybrid necrosis and other hybrid weakness were frequently observed in the F_1_ individuals and their progeny populations. In this study, we performed the phenotypic characterization and gene mapping of hybrid necrosis, with the aim of providing a basis for map-based cloning and elucidating the molecular mechanisms of the hybrid necrosis gene, which likely originated from *H. villosa*.

## Materials and methods

### Plant materials and growth conditions

Different accessions of *H. villosa* (2*n* = 2*x* = 14, VV) and *T. durum* (ZY1286, 2*n* = 4*x* = 28, AABB) were introduced and maintained at the Cytogenetic Institute of Nanjing Agricultural University (CINAU) and were designated with the starting character ‘CI’. We crossed tetraploid durum wheat ZY1286 (AABB) as the male parent and different accessions of diploid *H. villosa* (VV) as the female parents, and the obtained triploid F_1_ plants (ABV) were designated with the starting character ‘SH’. The F_1_ seeds were germinated and subsequently planted in the field during the growing season. After generating 3–5 tillers, the seedlings were removed from the soil and their roots were washed with clean water. Then the young stems on each tiller were punctured with anatomical needles and treated with 0.05% colchicine solution for 15–20 h at room temperature to induce chromosomal doubling. After the treatment, the seedlings were repeatedly washed with clean water and then transplanted into pots. The seeds from the S_0_ generation were harvested from surviving F_1_ plants. Further identification was carried out by genomic in situ hybridization (GISH) and molecular marker analyses to detect each chromosome. Through this process, multiple durum wheat*–H. villosa* amphiploids were successfully developed and they were designated with the starting character ‘STH’. An F_2_ mapping population and its F_2:3_ families were created by crossing two amphiploids STH59-1 and STH59-2. These materials were cultivated at the Baima Experimental Station of Nanjing Agricultural University, Nanjing, China, where they were grown in 1.2-m rows, spaced 25 cm apart, with 5 seeds planted per row in the field.

### Characterization of hybrid necrosis

During the adult stage, the phenotypes, including *H. villosa* CI084, *T. durum* *cv*. ZY1286, the newly developed multiple triploids, multiple amphiploids and the F_2_ population, were observed, and the photographs of hybrid necrosis were taken. A total of six amphiploids (STH75-3, STH73-3, STH77-4, STH79-2, STH50-3 and STH76-3) derived from different *H. villosa* accessions were selected for phenotypic observation throughout the entire growth cycle. The necrotic areas of the leaves were measured using ImageJ software (Abràmoff et al. 2004). During the grain filling stage, the degree of necrosis was assessed based on the necrotic area of the top-second leaf from the main tiller using a numerical scale of 0, 1 and 2. Grade 0 represented no (0%) necrosis; Grade 1, with a chlorosis value less than 40%, indicated weak necrosis with sporadic yellow necrotic spots; Grade 2, with a chlorosis value exceeding 40%, represented strong necrosis with severe yellowing and even withering. Individuals with different degrees of necrotic symptoms in the F_2_ population were selected for the investigation of agronomic traits, including plant height, tiller number, spike length, spikelet number per spike, thousand kernel weight, grain length and grain width. Statistical analysis was conducted through Duncan’s test, and the data were plotted using GraphPad Prism (V9.0.0).

### Cytogenetic analysis

To identify and characterize the chromosomes of *H. villosa* in the amphiploids STH59-1 and STH59-2, GISH and fluorescence in situ hybridization (FISH) were performed following Du et al. (2016), using cells during mitotic metaphase in the root-tips with amiprophos-methyl (APM) soaking and nitrous oxide treatment as described by Komuro et al. (2013). Total genomic DNA of *H. villosa* (91c43) labeled with 5’ FAM was used as the GISH probe, which produced green fluorescence signals in the V-genome chromosomes. Oligo-FISH was performed as described by Wang et al. (2017), using pAs1-1, pAs1-3, pAs1-4, pAs1-6, AFA-3, AFA-4, pSc119.2–1 and (GAA)10 as mixed probes to identify the different chromosome arms of the amphiploid. The hybridized slides were observed using an Olympus BX60 fluorescence microscope with a CCD camera DP72 (Olympus, Tokyo, Japan) for image acquisition. Individual chromosomes with hybridization signals were cropped using the Adobe Photoshop software (Adobe Systems, San Jose, CA, USA).

### Genetic analysis and gene mapping of *Ne-V*

The phenotypes of leaf necrosis in the F_2_ population were assessed and classified as Grades 0, 1 and 2. The genetic analysis of the goodness of fit test for a Mendelian ratio was performed with a Chi-square (χ2) test.

Genomic DNA was extracted from the leaf samples of each plant using a Plant Genomic DNA Extraction Kit (DP205, TianGen, China). Thirty plants with severe necrosis and 30 plants with no necrosis were selected according to their corresponding phenotypes in the F_2:3_ population to construct a severe-necrosis bulk and a no-necrosis bulk with an equal amount of DNA, respectively. The two DNA bulks were subjected to wheat exome capture sequencing by Mol Breeding Company, Shijiazhuang, China. The reference genomes of *T. durum* ‘Svevo’ (Maccaferri et al. 2019) and *H. villosa* ‘91c43^DH^’ (Zhang et al. 2023) were merged to form a reference genome for amphiploids (2*n* = 6*x* = 42, AABBVV). Fastp (v0.12.4) was used to clean and trim the raw data to remove low-quality reads and adapters. BWA (v0.7.17) was used to align the reads to the reference genome with the default settings. The BAM files were sorted using SAMTools (v1.6). GATK (v4.2.0) was utilized for PCR-duplicates and SNP calling, and BCFtools (v1.9) was used to filter each VCF file by “QD < 2.0 || FS > 60.0 || MQ < 40.0 || DP < 5”. Finally, the R package QTLseqR (Mansfeld and Grumet 2018) was used to calculate average G' values, using a 30 Mb sliding window.

For EST marker development, ESTs mapped on the long arms of wheat homoeologous group 2 chromosomes were retrieved from GrainGenes (www.graingenes.org), and those ESTs with no paralogous genes on other homoeologous chromosomes were selected for the design of primer pairs using the online software Primer 3 (https://bioinfo.ut.ee/primer3-0.4.0/). The 500-bp flanking sequences upstream and downstream of the SNP and the InDel sites were extracted from the *H. villosa* RefSeq (Zhang et al. 2023). The SNP markers were designed using the online software Primer1 (http://primer1.soton.ac.uk/primer1.html), and the InDel markers were designed using the PrimerServer of WheatOmics (Ma et al. 2021). Two EST-, one SNP- and four InDel markers (Table S6) were screened for polymorphisms between STH59-1 and STH59-2. STH59-2, with Grade 2 necrosis symptoms, was marked as ‘A’, and STH59-1, with Grade 0 necrosis symptoms was marked as ‘B’. The phenotypes of the F_2_ plants, whose individuals in the corresponding F_2:3_ lines showing Grade 2 necrosis symptoms, were marked as ‘A’; the F_2_ plants, whose individuals in the corresponding F_2:3_ lines showing Grade 0 necrosis symptoms, were marked as ‘B’; and the F_2_ plants, whose individuals in corresponding F_2:3_ lines showing segregated necrosis symptoms, were marked as ‘H’. A genetic map was constructed using the software Join-Map v4.0 (Van Ooijen 2006). Recombination frequencies were converted to centimorgans (cM) using the Kosambi mapping function (Kosambi 1944).

### Histochemical observation and physiological indices assay

At the heading stage, the leaves of STH59-1 and STH59-2 at the same leaf position were stained with diaminobenzidine (DAB) solution (1 mg/ml, pH 3.8) or Trypan blue (4 mg/ml). They were then decolorized in ethanol/acetic acid (3:1) for observation and photo acquisition. The H_2_O_2_ contents were measured during the heading stage using commercial kits (Jiancheng Biotechnology, Nanjing, China). The enzyme activities of peroxidase (POD), malondialdehyde (MDA) and superoxide dismutase (SOD) in the corresponding leaves were measured using enzyme detection kits (Comin Biotechnology, Suzhou, China). The experiments were carried out according to the manufacturer’s instructions.

### Measurement of photosynthetic pigment contents

During the heading stage, leaves with 0% necrosis from STH59-1 and leaves with 0%, ~50% and ~90% necrosis from STH59-2 were sampled for measurement of photosynthetic pigment contents via spectrophotometry. Three biological replicates were used for each sample. The calculation of the chlorophyll (Chl *a* and Chl *b*) and carotenoid (Car) contents in each leaf sample was based on Wellburn’s description (1994).

### Observation of chloroplast ultrastructure by transmission electron microscope

The chloroplast ultrastructure was observed by sampling normal leaves and severely necrotic leaves of STH59-1 and leaves of STH59-2. The processed specimens were examined and photographed using a transmission electron microscope (Hitachi H-7800, Tokyo, Japan).

### RT‒PCR analysis

Leaves of STH59-1 plants with no necrosis and leaves of STH59-2 plants with 0%, ~50% and ~90% necrosis were individually collected for RNA extraction with TRIzol total RNA extraction kits (Invitrogen, USA). cDNA was synthesized using the HiScript II Q RT Super Mix for qPCR (Vazyme Biotech, Nanjing, China). RT‒PCR was performed to analyze the expression level of defense related genes in leaves using gene-specific primers with *Tatubulin* as an internal control (Table S6).

### Analysis of the influence of light and temperature on leaf necrosis

To test the effect of light on necrosis, leaves of STH59-1 and STH59-2 at the mature stage were shaded with aluminum foil in pots in the greenhouse with 16-h light/8-h dark at 22 °C/18 °C day/night and 60–80% relative humidity in the commercial soil (70% peat soil, 15% vermiculite and 15% quartz sand). To investigate whether temperature has influence on necrosis, 15 seedlings of STH59-1 and STH59-2 were transferred to the growth chamber under 16-h light/8-h dark at constant temperatures of 14 °C, 23 °C or 30 °C, respectively. The initiation time and severity of necrosis were observed and recorded.

## Results

### Phenotypic characterization of triploid F_1_ plants derived from *T. durum *and *H. villosa* crosses with hybrid necrosis

Independent crosses were previously made between *T. durum* *cv*. ZY1286 and 13 *H. villosa* accessions. In total, 83 triploid F_1_ plants were produced from all the *H. villosa* accessions. Based on field observations, we found that 58 F_1_ plants derived from 12 *H. villosa* accessions exhibited hybrid necrosis. These plants exhibited typical leaf chlorosis, yellowing and necrotic patches throughout the growth period. This indicates that 58 out of 83 (69.9%) F_1_ plants, or 12 out of 13 (92.3%) *H. villosa* accession-derived progeny, exhibited hybrid necrosis (Fig. S1). However, the leaves of the hybrid parents remained green and only some basal leaves turned yellow normally during the maturation stage (Fig. S2). The ratio of the chlorosis area of the top-second leaf from the triploid F_1_ plants was calculated to measure the severity of hybrid necrosis. Then, symptoms of each F_1_ individual were classified as Grade 0–2 according to their severity. The results indicated that F_1_ plants derived from *H. villosa* accessions CI076, CI079, CI085, CI087, CI092, CI093, CI096, CI106, CI108 and CI111 exhibited no necrosis (Grade 0), those derived from *H. villosa* accessions CI093, CI097, CI106 and CI109 exhibited Grade 1 necrosis, and those derived from *H. villosa* accessions CI074, CI076, CI079, CI087, CI092, CI096, CI097, CI108, CI109 and CI111 exhibited Grade 2 necrosis (Table S1). It was also found that different F_1_ plants derived from the same *H. villosa* accession, including accessions CI076, CI079, CI087, CI092, CI093, CI096, CI097, CI106, CI108, CI109 and CI111, displayed varying degrees of hybrid necrosis. This might be caused by the high genetic heterogeneity of *H. villosa* due to open pollination.

### Phenotypic analysis of hybrid necrosis in hexaploid amphiploid between *T. durum* and *H. villosa*

**Fig. 1:**
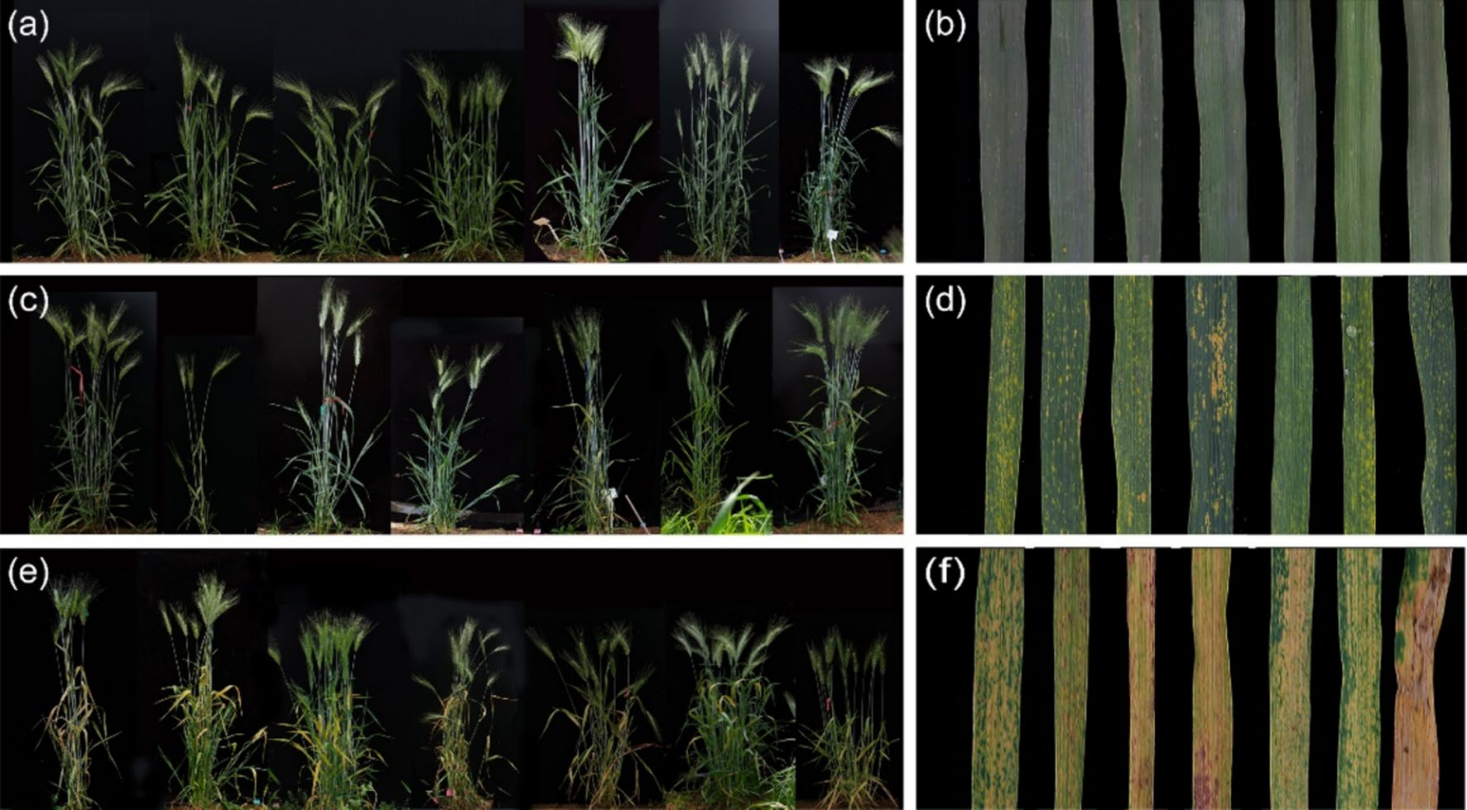
The whole plants and the appropriate leaves phenotypes of the synthetic amphiploid lines (AABBVV) of durum wheat–*H. villosa* at the adult stage **a, b** The whole plants and leaves presented normal phenotypes. **c, d** The whole plants and leaves presented moderate necrotic phenotypes. **e, f** The whole plants and leaves presented severe necrotic phenotypes

*T. durum*–*H. villosa* amphiploids (2*n* = 6*x* = 42, AABBVV) were developed as the bridge parents for transferring *H. villosa* chromatin to common wheat. We observed that 22 amphiploids, including the newly developed amphiploids from 21 *H. villosa* accessions and one amphiploid originating from *H. villosa* accession 91c43, exhibited different phenotypes of leaf necrosis. Similar to the leaf necrosis phenotypes observed in the F_1_ individuals, the leaf necrosis phenotypes of the 78 amphiploids were classified into three grades. Grade 0 corresponded to the amphiploids derived from *H. villosa* accessions CI054, CI058, CI062, CI071, CI075, CI077, CI080, CI084, CI086, CI090, CI091, CI100, CI102 and CI104; Grade 1 corresponded to the amphiploids derived from *H. villosa* accessions 91c43, CI061, CI071, CI075, CI077, CI080, CI086, CI088, CI090, CI091, CI094, CI098, CI099 and CI103; and Grade 2 corresponded to the amphiploids derived from *H. villosa* accessions CI075, CI080, CI084, CI098, CI099, CI100, CI101, CI102, CI103 and CI104. In addition, the amphiploids derived from 15 *H. villosa* accessions displayed different types of hybrid necrosis with distinct segregation, which was caused by heterogeneity of *H. villosa* (Table S2, Fig. 1). In this study, we found that 48 T*. durum–H. villosa* amphiploids among 78 amphiploids showed hybrid necrosis, indicating that 48 out of 78 (61.5%) amphiploids, or 19 out of 22 (86.4%) *H. villosa* accessions-derived progeny exhibited hybrid necrosis.

### Hybrid necrosis of the amphiploids derived from different *H. villosa* accessions showed distinct developmental processes related to growth stage

Six amphiploids with the same Grade 2 leaf necrosis derived from *H. villosa* accessions CI098, CI100, CI101, CI102, CI104 and CI075, were selected for a detailed comparison of the developmental processes of hybrid necrosis during the whole growth period. The results indicated that leaf necrosis in different amphiploids occurs at different growth stages. Among the six amphiploids, two (STH75-3 and STH73-3) showed necrosis during the tillering stage with 2–3 tillers, three (STH77-4, STH79-2 and STH76-3) showed necrosis during the tillering stage with 5–8 tillers, and one (STH50-3) showed necrosis during the booting stage (Fig. S3). In addition, the development processes of the necrosis showed different patterns. For example, necrosis in STH75-3 and STH77-4 occurred rapidly from the tillering stage to the jointing stage, with two-thirds of the basal leaves being severely necrotic during the booting stage. Necrosis in STH73-3 and STH79-2 progressed slowly during the tillering stage, with one-third of the basal leaves showing slight necrosis during the booting stage. Only a few basal leaves of STH76-3 experienced slight yellowing and necrosis before and during the booting stage, while almost all the leaves on the main stem experienced extensive necrosis during the filling stage. However, STH50-3 had some dead basal leaves, while the remaining leaves showed slight necrosis from the tillering to the filling stage (Fig. S4). The severity of necrosis on the top of the second leaves on the main stem of the amphiploids described above was evaluated. The results indicated that STH73-3, STH75-3, STH76-3, STH77-4 and STH79-2 exhibited severe necrosis (Grade 2), while STH50-3 showed weak necrosis (Grade 1) (Fig. S5). Due to the amphiploids sharing the same tetraploid parent, ZY1286, the variation in leaf necrosis detected among amphiploids is likely due to necrosis-related genes at different loci or to necrosis-related genes with different effects at the same loci in different *H. villosa* accessions.

### Development of *T. durum*–*H. villosa* amphiploids with significantly different necrosis phenotypes

**Fig. 2:**
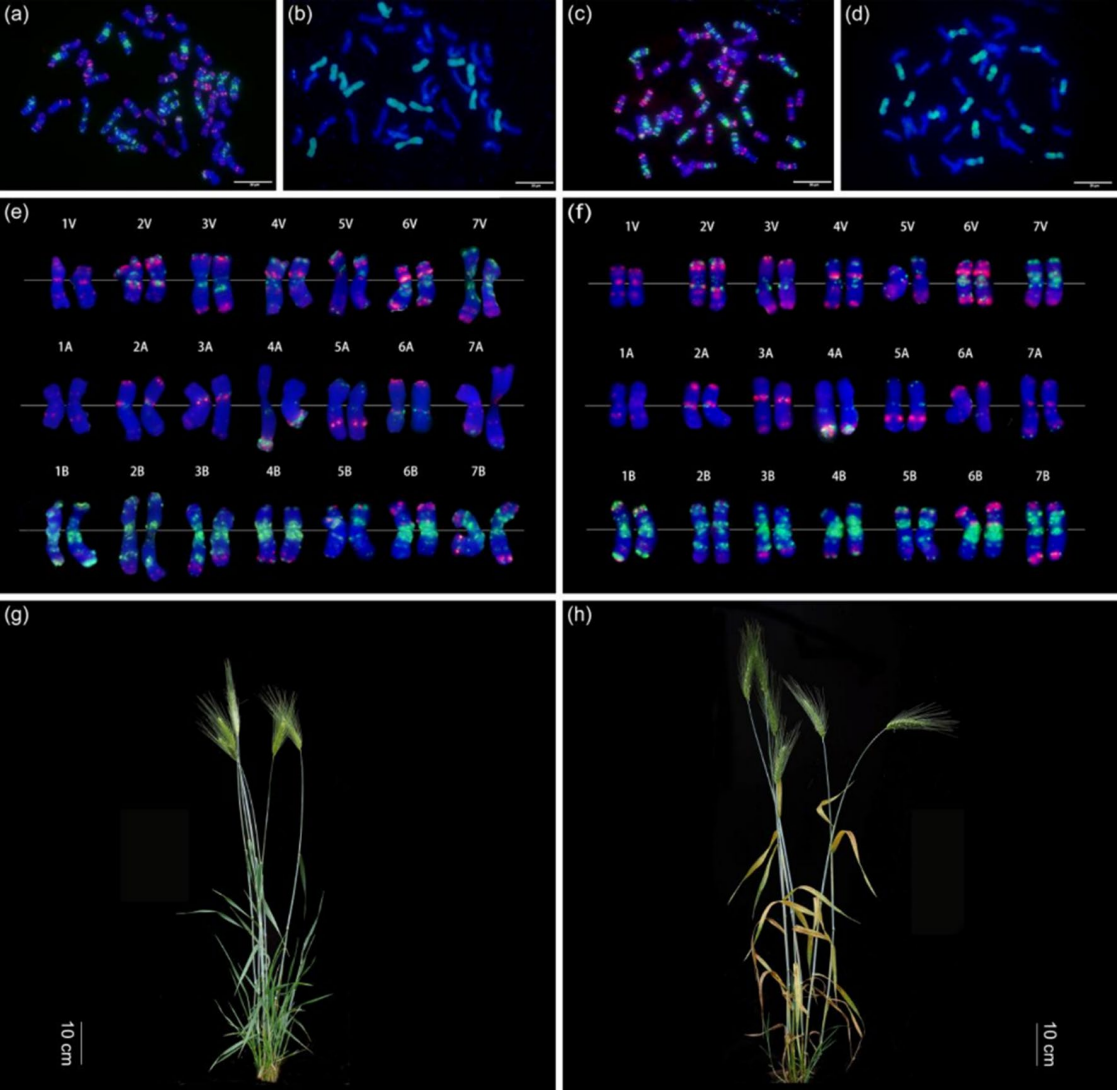
Cytogenetic identification and the whole plant phenotypes of the newly synthesized amphiploids (AABBVV) STH59-1 and STH59-2 **a–d** Fluorescence in situ hybridization (FISH) and genomic in situ hybridization (GISH) analysis of amphiploids (AABBVV) STH59-1 (**a**, **b**) and STH59-2 (**c**, **d**) on root tip metaphase chromosomes. The punctate red and green fluorescence signals in the FISH analysis were obtained using mixed oligo probes, which allowing identification of individual chromosomes. The green fluorescence in GISH analysis represents signals obtained using the total genomic DNA of *H. villosa* as probe. Chromosomes were counterstained with 4’, 6-diamidino-2-phenylindole (DAPI) and fluoresced blue. **e, f** Karyotypes of the A, B, V-genome chromosomes of the two amphiploids. The paired chromosomes were extracted from **a** and **c,** respectively. **g, h** The whole plant phenotypes of normal STH59-1 and necrotic STH59-2 at the adult stage

The *T. durum*–*H. villosa* amphiploids, STH59-1 and STH59-2, with no or weak or severe grades of necrosis, respectively, were obtained by wide hybridization between tetraploid *T. durum cv.* ZY1286 (AABB) and diploid *H. villosa* CI084 (VV) (Fig. 2). The results of GISH and Oligo-FISH analysis revealed that both STH59-1 and STH59-2 contained 42 chromosomes including 28 chromosomes from *T. durum* and 14 chromosomes from *H. villosa* (Fig. 2). Oligo-FISH showed that the florescence signals on the A- and B-genome chromosomes were similar between STH59-1 and STH59-2. However, slight differences were observed in the florescence signals of the V-genome chromosomes, such as those on the long arm of 4V and the short arm of 5V. Agronomic trait identification revealed no difference between STH59-1 and STH59-2 in terms of plant height, effective tillers or spike length. However, a significant difference was observed in spikelet number (Table S3).

### Characterization of hybrid necrosis in STH59-2

In the greenhouse, necrosis in the fourth leaf of STH59-2 started at the tip area during the four-leaf stage. Afterward, the necrosis spread from the tip to the bottom during the tillering stage (Fig. S6). With the development of STH59-2 plants, necrosis spread from the older bottom leaves to the younger upper leaves, extending from the tip to the base of the leaf, and even resulting in the withering of the whole plant (Fig. S7a). However, the leaves of STH59-1 remained green during all the developmental stages under the same conditions (Fig. S7b). The extent of necrosis spreading on a single tiller was also clear, as described above (Fig. S7c–d).

Leaf necrosis is usually correlated with a decrease in chlorophyll content. We measured the chlorophyll and carotenoid contents in leaves with 0%, ~50% or ~90% necrotic ratios of STH59-2 and in the normal leaves of STH59-1 (control). The contents of both chlorophyll a and chlorophyll b decreased in the leaves with a necrotic ratio of~50% or ~90%, respectively, compared to those with a necrotic ratio of 0% in the STH59-2 leaves. Similarly, the carotenoid content decreased gradually with increasing necrosis severity in the STH59-2 leaves. The normal leaves of STH59-2 and STH59-1 had no differences in chlorophyll a or carotenoid content; however, the normal leaves of STH59-2 had a decrease in chlorophyll b content compared to the normal leaves of STH59-1 (Fig. S8).

**Fig. 3:**
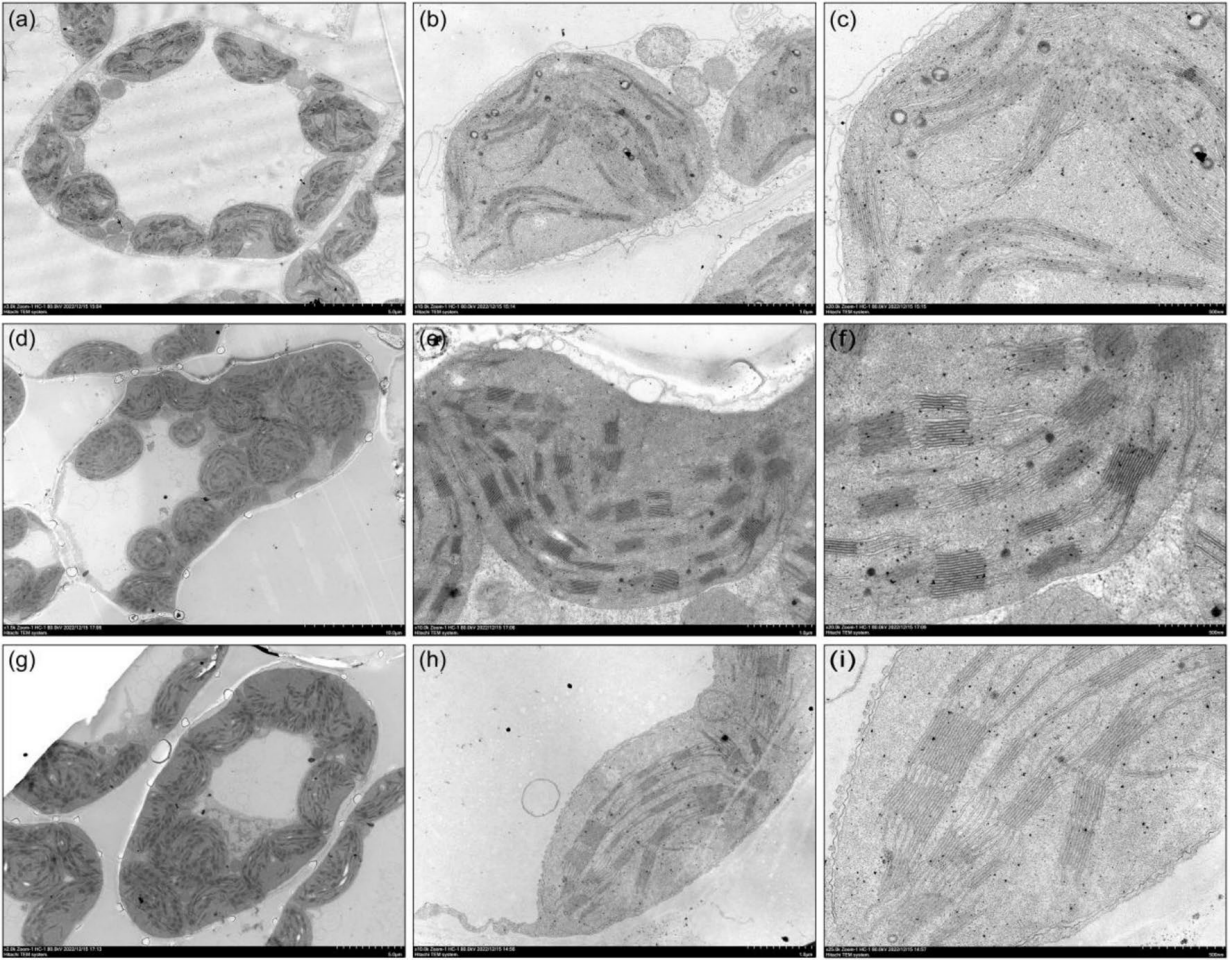
Transmission electron micrographs of the intracellular structure in the necrotic and normal leaves detached from STH59-2 and STH59-1 **a–c** Mesophyll cells in necrotic leaves of STH59-2 showed changed chloroplasts with disordered or degraded lamellar structure and vacuolated mitochondria. **d–i** Mesophyll cells in normal leaves of STH59-2 (**d, f**) and STH59-1(**g–i**) showed well-ordered chloroplasts with orderly arranged lamellar structure and intact mitochondria. Scale bar: 5 μm in **a** and **g**, 10 μm in **d**, 1 μm in **b**, **e** and **h**, 500 nm in **c**, **f** and **i**

**Fig. 4:**
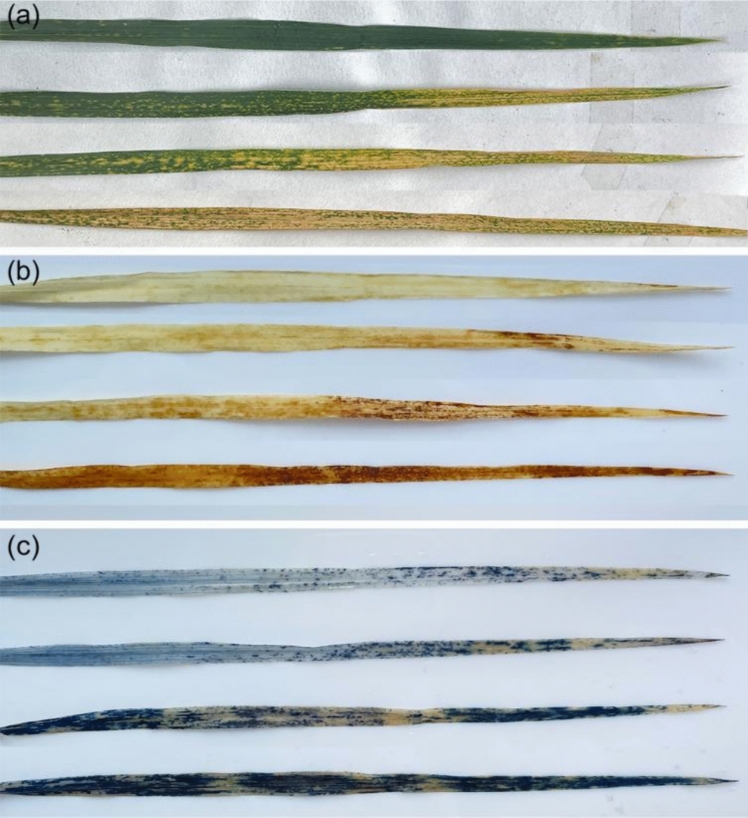
Phenotypic characterization of hybrid necrosis in STH59-2 **a–c** From top to bottom are the leaves in same leaf position sampled from the necrotic STH59-2 plants classified with different necrotic degrees, which were unstained leaves (**a**) DAB stained leaves (**b**) and Trypan blue stained leaves (**c**). It indicated that H_2_O_2_ accumulating (**b**) and cell death (**c**) increased significantly in the leaf area with serious necrosis symptom (**a**)

The chloroplast structure was observed to be normal with orderly arranged lamellar structures and intact mitochondria in the normal leaves of STH59-1 and STH59-2. However, the chloroplast structure in the necrotic leaves of STH59-2 changed, with disordered or degraded lamellar and vacuolated mitochondria (Fig. 3). Subsequently, the detached top one to the top fourth leaf of the same tiller in STH59-2 and STH59-1 plants were separately stained with DAB or Trypan blue for the histological detection of the physiological state of the cells. It was also found that both H_2_O_2_ accumulation and cell death increased significantly in the leaf area with severe necrosis (Fig. 4).

### Genetic analysis and mapping of the hybrid necrosis gene *Ne-V* in STH59-2

**Fig. 5:**
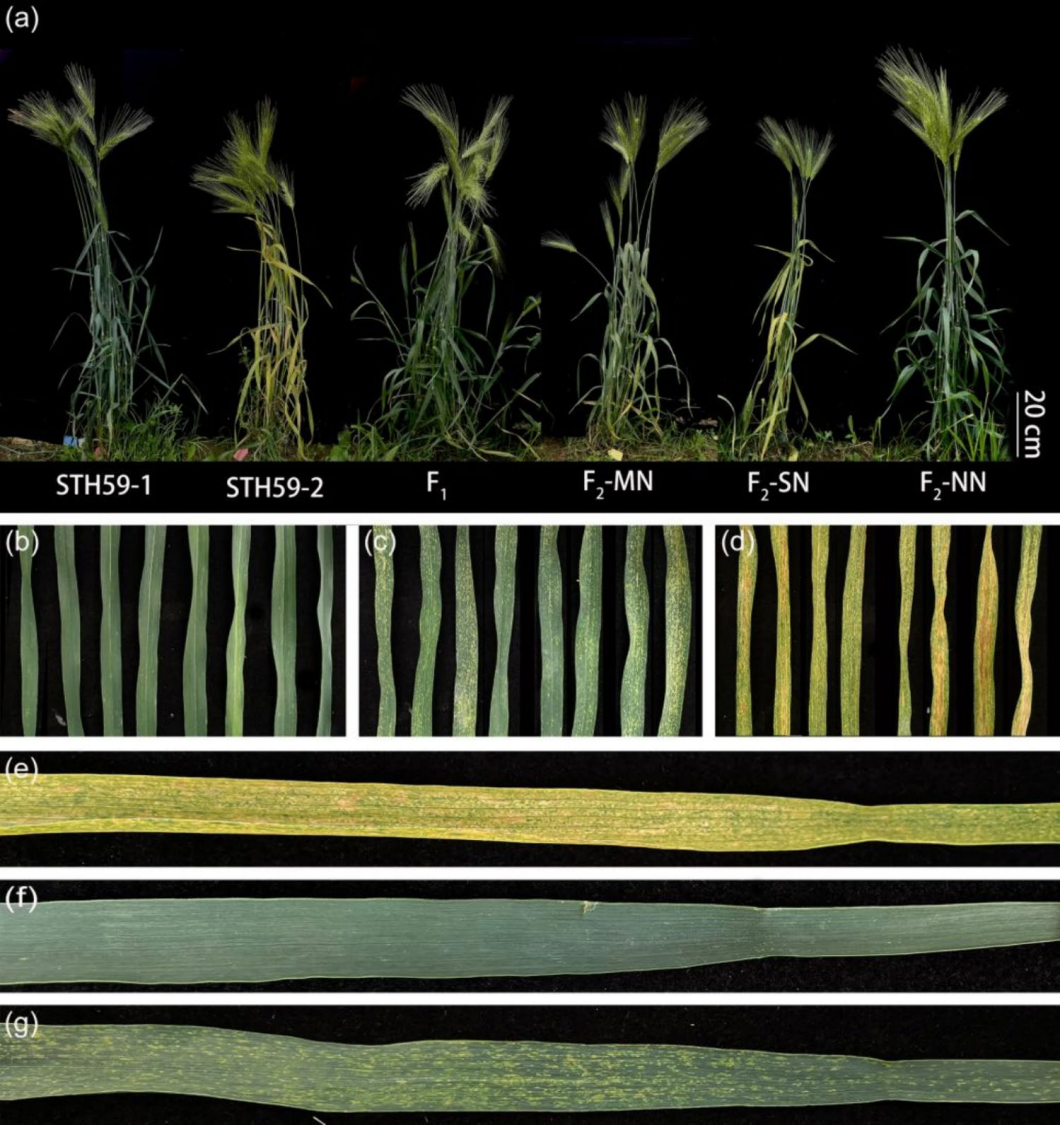
Phenotypes of STH59-1, STH59-2 and their derived F_1_ individual and F_2_ population **a** STH59-1 is normal parents, STH59-2 is severe necrotic parents, F_1_ is the STH59-1/STH59-2 cross derived F_1_ individual with moderate necrosis symptom, F_2_-MN is the derived F_2_ individual with type II moderate necrosis symptom, F_2_-SN is the derived F_2_ individual with type III severe necrosis symptom, F_2_-NN is the derived F_2_ individual with type I non-necrosis symptom. **b** Leaf photographs of F_2_-NN individuals. **c** Leaf photographs of F_2_-MN individuals. **d** Leaf photographs of F_2_-SN individuals. **e–g** Leaf photographs of the parents STH59-2, STH59-1 and their cross derived F_1_ individual

To identify the hybrid necrosis gene in STH59-2 with the greatest degree of necrosis (91.4%), we created a mapping population by crossing STH59-2 with STH59-1 without hybrid necrosis. Hybrid necrosis symptoms were less severe in the F_1_ plants than in STH59-2 plants. The F_2_ plants were divided into three groups, 100 Grade 0 plants without necrosis, 153 Grade 1 plants with moderate necrosis and 91 Grade 2 plants with severe necrosis (Fig. 5). The statistical analysis showed that the separation ratio was 1:2:1 (*χ*^2^ = 4.669, *P* = 0.0969), which is suitable for single-gene segregation. This indicated that the hybrid necrosis symptom was putatively controlled by a semidominant gene and was tentatively designated as *Ne-V*. All the individuals of the F_2:3_ lines derived from F_2_ plants with Grade 0 leaf symptoms showed no necrosis, and those derived from F_2_ plants with Grade 2 leaf symptoms showed the same severe necrosis, while those derived from F_2_ plants with Grade 1 leaf symptoms segregated in necrosis symptoms, including Grade 0, Grade 1 and Grade 2 leaf symptoms. These results further suggested that the hybrid necrosis of STH59-2 was controlled by the semidominant gene *Ne-V* (Fig. S9).

**Fig. 6:**
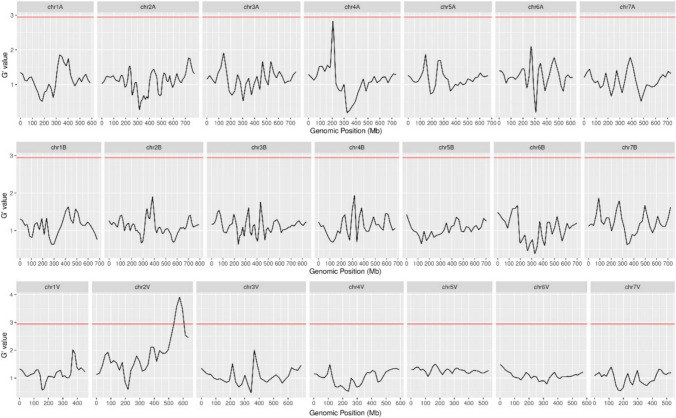
Identification of gene loci related to hybrid necrotic gene *Ne-V* by G’ value

The bulked segregant analysis coupled with exome capture sequencing (BSE-Seq) generated approximately 14.48 Gb of necrotic pool and 13.94 Gb of the non-necrotic pool (Table S4). Compared to the reference genome of the amphiploids, 96.48% of the reads in the necrotic pool and 96.70% of the reads in the non-necrotic pool could be aligned with the reference genome (Table S5). By analyzing the smoothed G statistics (G’) value within a window size of 30 Mb genomic region window across the genome, only one candidate genomic region strongly associated with the necrosis phenotype was identified in a 71-Mb region ranging from 535.33 Mb to 606.34 Mb on the long arm of chromosome 2V (Fig. 6). There were 14,012 SNP markers in this interval, with 197 markers per Mb.

**Fig. 7:**
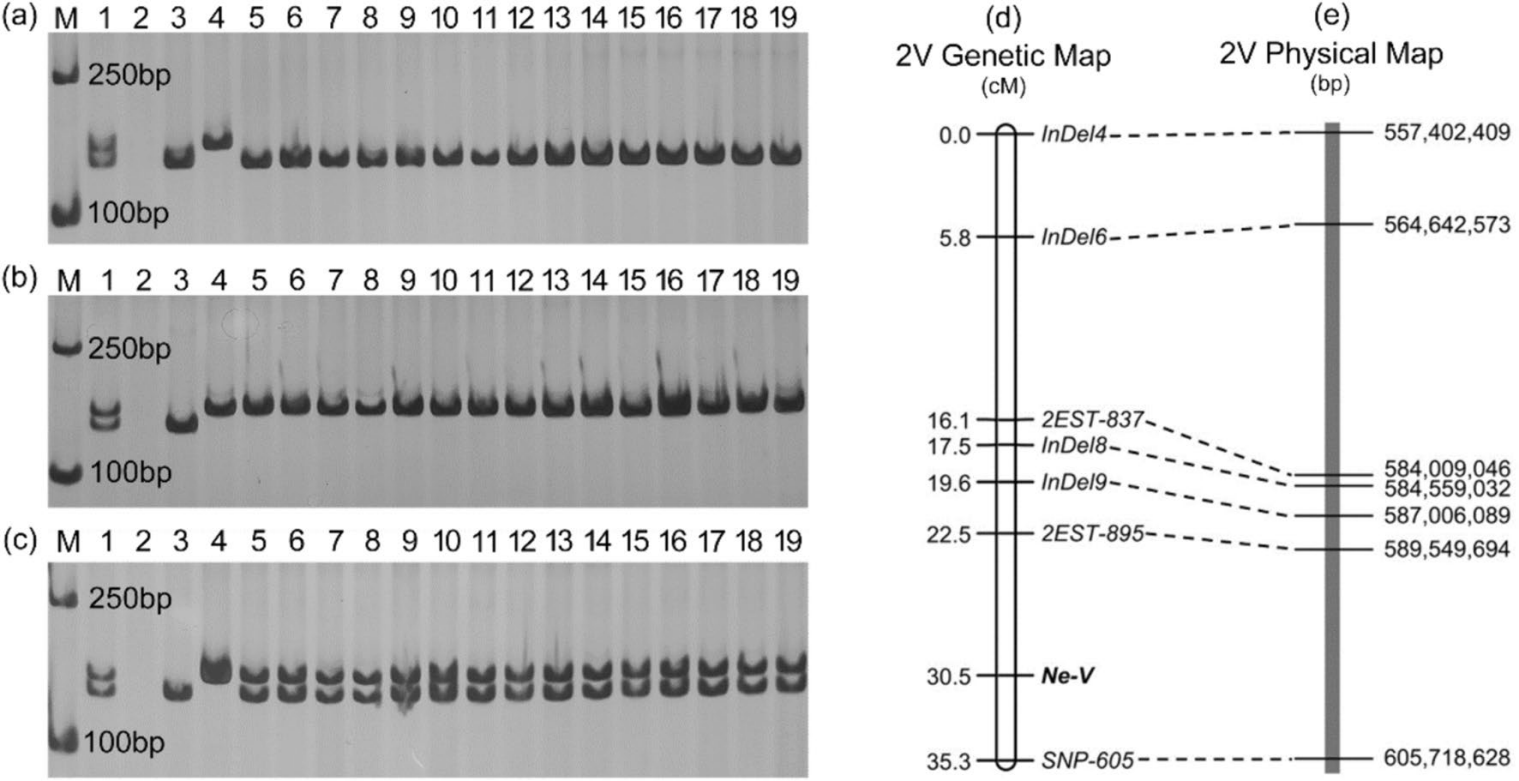
Genetic linkage map for *Ne-V* on chromosome 2V **a–c** *InDel9* marker tested in the STH59-1/STH59-2 and their derived F_2_ population individuals. M, 1, 2, 3 and 4 represent DNA Maker DL2000 (TAKARA, Japan), CI084, ZY1286, STH59-1 and STH59-2, respectively. 5-19 lanes represented F_2_ plants with Grade 0 non-necrosis symptom (**a**), F_2_ plants with Grade 2 severe necrosis symptom (**b**) and F_2_ plants with Grade 1 moderate necrosis symptom (**c**). **d** Genetic linkage map of hybrid necrotic gene *Ne-V*, genetic distances (cM) and markers are shown on the left and right, respectively. **e** The physical positions (bp) of the polymorphic linkage markers on 2VL of *H. villosa* RefSeq are indicated on the right

Subsequently, the markers from seven chromosomes of *H. villosa* were used to analyze the F_2_ population, and it was found that the polymorphic markers on chromosome 2V, but not on other chromosomes, were genetically linked to *Ne-V*, further indicating that *Ne-V* is located on chromosome 2V. Seven molecular markers that show polymorphisms between STH59-2 and STH59-1 were developed, including five markers developed based on the SNP and InDel information obtained from BSE-Seq and two EST markers developed from the homoeologous group 2 chromosomes of wheat. The F_2_ individuals were genotyped using seven co-dominant polymorphic markers, and a local genetic map covering the *Ne-V* locus was constructed. This map consisted of seven marker loci spanning 35.3 cM. The *Ne-V* gene could be mapped to the long arm of chromosome 2V, and it was closely linked to the markers *2EST-895* and *SNP-605* with genetic distances of 7.0 cM and 4.8 cM, respectively (Fig. 7d), corresponding to a 16.17 Mb interval in *H. villosa* RefSeq (Fig. 7e).

### Effects of hybrid necrosis on agronomic traits

The agronomic traits of individual plants exhibiting varying degrees of leaf necrosis, including those with severe, moderate and no necrosis, in the F_2_ population were investigated. Necrosis had no significant effect on the growth or development of the plants. No significant differences were found among the three types of F_2_ plants in terms of plant height, tiller number, spike length or spikelet number (Fig. S10). However, severe necrosis had a significant negative effect on grain characteristics such as grain length, grain width and thousand grain weight. These parameters were significantly lower in plants with severe necrosis than in those with moderate or no necrosis (Fig. S11).

### Mechanism of hybrid necrosis

Plant hybrid necrosis has been unequivocally linked to autoimmunity, and it is genetically and biochemically similar to the hypersensitive response (HR) associated with pathogen resistance. Therefore, the expression levels of several marker genes in the defense signaling pathway were analyzed in the normal leaves of STH59-1 and the leaves of STH59-2 with 0%, ~50% and ~90% necrosis by RT‒PCR. As shown in Fig. S12, the pathogenesis-related (PR) genes, including *PR1*, *PR2*, *PR3* and *PR10*, were exclusively expressed in the necrotic leaves of STH59-2, while no expression was detected in the normal leaves of STH59-1 and STH59-2.

Expression analysis of the antioxidant genes revealed that the catalase (*CAT*) and peroxidase (*POD*) genes were obviously induced by necrosis, while superoxide dismutase (*SOD*), ascorbate peroxidase (*APX*) and glutathione-S-transferase (*GST*) were expressed in both normal and necrotic leaves. This result indicated that the defense response might have changed when necrosis occurred. Additionally, the contents of malondialdehyde (MDA) and H_2_O_2_, as well as the enzyme activity of POD and SOD, were tested in the severely necrotic leaves of STH59-2 and normal leaves of STH59-2 at the same leaf position. The contents of MDA and H_2_O_2_, as well as the activity of POD, in the severely necrotic leaves of STH59-2 were significantly greater than those in the normal leaves of STH59-2, while the activity of SOD showed no significant difference between both materials (Fig. S12). These results indicated that H_2_O_2_ and POD accumulated during the process of necrosis.

**Fig. 8:**
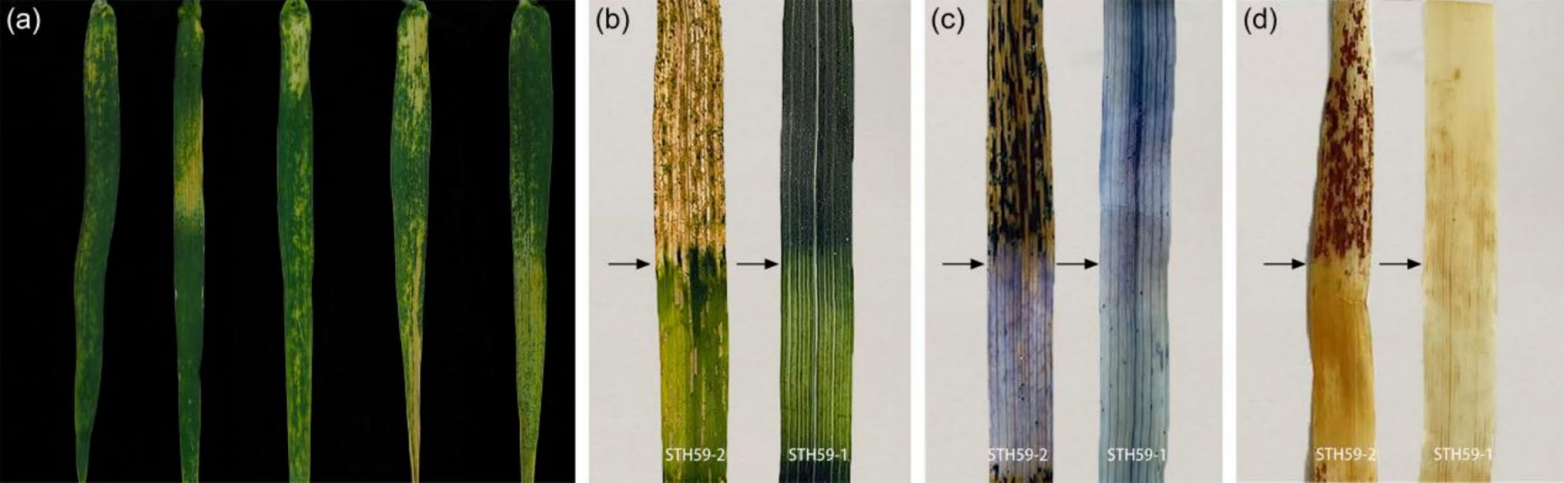
The effect of illumination and shading treatment on hybrid necrosis **a** Uneven illumination causes yellowing and necrosis of STH59-2 leaves from different positions. **b–d** The leaves of STH59-2 and STH59-1 at the same leaf position were partially shaded at the mature stage. For each leaf, the upper part was the normal growth area, and the lower part is the shaded area. In leaves of STH59-1, no necrosis was observed in both shading and illuminating area (**b**). No cell death and no hydrogen peroxide accumulation were detected after Trypan blue staining (**c**) and DAB staining (**d**). In leaves of STH59-2, necrosis (**b**), cell death (**c**) and hydrogen peroxide accumulation (**d**) were only detected in illuminating area but not in shading area

In the field, we found that necrosis extended from the leaf tip to the base of the leaf. However, necrosis initiated at different locations under the controlled conditions in the growth chamber. It was hypothesized that environmental conditions play an important role on inducing necrosis. Therefore, we conducted a shading experiment to measure the impact of light on necrosis initiation. The leaves of STH59-2 and STH59-1 at the same leaf position were partially shaded during the mature stage and they were evaluated 14 days later. In the leaves of STH59-1, no necrosis was observed in either the shaded or illuminated areas, and no cell death or hydrogen peroxide accumulation was detected histologically by DAB or Trypan blue staining. In the leaves of STH59-2, necrosis, cell death and hydrogen peroxide accumulation were detected in the illuminated area, but not in the shaded area (Fig. 8). In addition, temperature had a significant impact on the process of leaf necrosis. The necrosis of STH59-2 occurred earlier at 14 °C and delayed at 30 °C, suggesting that lower temperatures could accelerate leaf necrosis and that higher temperatures could delay it (Fig. S13). Therefore, illumination and temperature strongly influence the occurrence of leaf necrosis in STH59-2.

## Discussion

### Hybrid necrosis was widely detected between *T. durum* and multiple *H. villosa*

In the past decade, we have successfully developed 78 T*. durum–H. villosa* amphiploids using 22 *H. villosa* accessions. Most amphiploids showing hybrid necrosis attracted our interest in the research on necrotic genes in *H. villosa*. We then performed wide crosses between *T. durum* *cv*. ZY1286 and 13 other *H. villosa* accessions to synthesize amphiploids. The newly obtained F_1_ plants and amphiploids in the early stage were used for observation of hybrid necrosis symptoms. Comparisons of the grades of leaf necrosis between F_1_ plants and their corresponding amphiploids revealed that the necrosis grades remained consistent across generations, indicating that hybrid necrosis was stable and not affected by chromosome ploidy. We found that the progenies from 31 *H. villosa* accessions exhibited necrosis phenotypes, suggesting that 31 out of 35 (88.6%) of *H. villosa* accessions harbor hybrid necrosis-related genes.

In addition, the progenies from the same *H. villosa* accessions exhibited various necrosis phenotypes, and it is consistent with the high heterogeneity of *H. villosa*. Different pollens from the same accession of *H. villosa* carried different alleles of the hybrid necrosis gene, resulting in different hybrid necrosis phenotypes in their derived progenies. We also performed the genotypic analysis of STH59-1, STH59-2 and two donor lines (CI084 and ZY1826) using six polymorphic markers linked to *Ne-V*. It was found that two markers were heterozygous in CI084, while the other four markers were homozygous in CI084 as shown in Fig. S14. Therefore, we speculated that the *H. villosa* CI084 is heterozygous. This finding was consistent with the difference in the necrotic phenotype between STH59-1 and STH59-2 derived from CI084. The results also revealed the complex genomic composition of this locus due to the heterogeneity of *H. villosa* CI084.

### Different types of hybrid necrosis were detected in various *H. villosa*-derived progenies

In this study, hybrid necrosis was detected in amphiploids derived from *T. durum* and multiple accessions of *H. villosa*, suggesting that necrosis genes in *H. villosa* interact with the necrosis genes in *T. durum*. In bread wheat, there are multiple alleles at each locus of *Ne1* and *Ne2*. *Ne1* have three alleles: *Ne*_*1*_^*w*^, *Ne*_*1*_^*m*^ and *Ne*_*1*_^*s*^ for weak, moderate and strong hybrid necrosis, respectively, while *Ne2* have five alleles: *Ne*_*2*_^*w*^ (weak), *Ne*_*2*_^*mw*^ (moderate-weak), *Ne*_*2*_^*m*^ (moderate), *Ne*_*2*_^*ms*^ (moderate-strong) and *Ne*_*2*_^*s*^ (strong) (Hermsen 1963; Si et al. 2021a). It was found that different combinations of alleles at the *Ne1* and *Ne2* loci lead to the varying severities of hybrid necrosis. Similarly, in the hybridization between rye and bread wheat, different combinations of *Ner1* and *Ner2* in the R genome of rye with *Ne1* and *Ne2* in bread wheat can lead to different degrees of hybrid necrosis. In this study, different degrees of necrosis were observed in triploid F_1_ plants and amphiploid plants between *T. durum* and different accessions of *H. villosa*, indicating that different *H. villosa* accessions may carry different necrosis gene loci, or alleles with different necrosis effects at the same locus. This phenomenon needs to be investigated by genetic analysis using materials with different degrees of necrosis.

Hybrid necrosis genes, including *Ne1* and *Ne2* in bread wheat, *Net1* and *Net2* in synthetic hexaploid wheat, and *Ner1* and *Ner2* in rye, have been mapped in cereal crops. *Ne1*, *Ne2* and *Net2* are located on chromosome arms 5BL, 2BS and 2DS, respectively (Chu et al. 2006; Mizuno et al. 2011; Ren and Lelley 1988). Previous studies have shown that *Ne1* is expressed mainly in tetraploid wheat (Tsunewaki 1992; Vikas et al. 2013). We analyzed the *Ne1* in ZY1286 with the co-segregating marker *5B-InDel385*, which was a precise marker for the detection of *Ne1* (Si et al. 2021b) and found that *Ne1* was present in ZY1286 (Fig. S15). Wheat plants harboring *Ne1Ne1ne2ne2*, *ne1ne1Ne2Ne2* or *ne1ne1ne2ne2* grow normally. However, hybrid necrosis occurs when plants carry both *Ne1* and *Ne2* (*Ne1-Ne2-*) (Hermsen 1963). Multiple alleles at each locus of *Ne1* and *Ne2* lead to different combinations of *Ne* alleles, resulting in various degrees of hybrid necrosis in different crosses (Vikas et al. 2013). The genotype of the durum wheat *cv.* ZY1286 is *Ne1Ne1ne2ne2*, while the genotypes of some *H. villosa* accessions are *ne1ne1Ne-VNe-V* with multiple *Ne-V* alleles, and the genotypes of some accessions are *ne1ne1ne-Vne-V*. Therefore, the amphiploids showed hybrid necrosis in the *Ne1Ne1Ne-VNe-V* genotypes, while the grades of hybrid necrosis varied depending on the *Ne-V* allele.

In this study, *Ne-V* was located on 2VL, which belongs to the homoeologous group 2 chromosome, similar to *Ne2* and *Net2*. However, *Ne2* and *Net2* were located on the short arm, while *Ne-V* was located on the long arm. Collinearity analysis of the group 2 chromosomes revealed no rearrangement of the chromosome structure on the long arm of the chromosome 2V. Therefore, it was suggested that *Ne-V* was non-allelic to *Ne2* or *Net2* and may be a new member different from other necrotic genes.

### Hybrid necrosis was similar to the immune response

It has been reported that hybrid necrosis in different species is caused by uncontrolled activation of immune responses in the absence of pathogens. Kostoff (1930) proposed a link between hybrid necrosis and autoimmunity, pointing out that the phenotype of hybrid necrosis is similar to that caused by pathogens. This suggests that autoimmunity is the main cause of hybrid necrosis. Li and Weigel (2021) also reported that hybrid necrosis is an extreme result of immune system dysfunction caused by the combination of independently evolved immune components in different parental lineages.

In most cases, hybrid necrosis genes encode NLR proteins, which are the typical proteins of disease resistance genes. Some hybrid necrosis genes have been characterized as resistance genes. For example, *Ne2*^*m*^ (*Lr13*) is resistant to leaf rust and is subjected to positive selection during wheat breeding, *Cf-2* is resistant to *Cladosporium fulvum* in tomato, *Rin4* confers race-specific resistance in an interspecific lettuce hybrid, and *ACD6* improves disease resistance in *Arabidopsis* by regulating the salicylic acid pathway (Yan et al. 2021; Hewitt et al. 2021; Krüger et al. 2002; Jeuken et al. 2009; Todesco et al. 2014). The molecular mechanism of hybrid necrosis has been revealed to be similar to that of the immune response (Wang et al. 2018; Chen et al. 2016). When two independently evolved immune systems in each parent are combined in F_1_ plants, the immune system is easily over-activated due to the mismatch of components in the highly complex immune system of F_1_ plants (Si et al. 2021a). Therefore, F_1_ plants often exhibit a hybrid necrosis phenotype similar to a strong HR phenotype, a macroscopic demonstration of local cell death usually associated with plant defense against pathogens (Chae et al. 2014).

In this study, a hypersensitive response was observed in the necrotic plants and in the necrotic area along with superoxide accumulation and PR gene expression. These findings were consistent with those of Xue et al. (2015) and Si et al. (2021a) for other wheat species. We found that the MDA content, H_2_O_2_ content and POD activity of severely necrotic leaves of STH59-2 were significantly greater than those of normal leaves of STH59-1, indicating that hybrid necrosis is accompanied by severe oxidative stress reactions in plant cells. The main reactive oxygen species generated in cells, H_2_O_2_, induces the expression of POD genes in severely necrotic leaves of STH59-2 plants. However, excessive accumulation of H_2_O_2_ causes irreversible oxidative damage to cells leading to necrosis. It is speculated that after wide hybridization, the immune systems of *H. villosa* and the durum wheat "ZY1286" were in the same individual and the independently evolved immune components in the two species recombined, resulting in strong immune system dysfunction and an explosion of reactive oxygen species in leaf cells. Therefore, the occurrence of hybrid necrosis in amphiploids is typically similar to plant immune reactions. It is interesting to investigate whether the necrosis gene *Ne-V* carried by STH59-2 also confers resistance to a certain disease.

### Hybrid necrosis was strongly influenced by the environment

Because temperature is a key environmental factor affecting the intensity of the plant immune response, the phenotype of hybrid necrosis might change under different temperatures (Li and Weigel 2021). In artificially synthesized wheat, Type II necrosis is significantly induced by low temperature (Sakaguchi et al. 2016). In *Arabidopsis*, F_1_ individuals exhibit dwarfism and tissue necrosis at 16 °C, while severe necrosis is alleviated at 28 °C (Chae et al. 2014; Todesco et al. 2014). In contrast, the necrosis in the hybrid offspring of the rice variety “Teqing” and wild rice became more severe under relatively high temperatures, while the necrosis was alleviated under relatively low temperatures (Chen et al. 2014). In this study, higher temperatures inhibited the occurrence of necrosis, while lower temperatures accelerated the occurrence of necrosis, indicating that the hybrid necrosis in STH59-2 is also affected by temperature.

Previous studies have shown that necrosis symptoms can be alleviated under short day conditions, indicating that light has a certain impact on hybrid necrosis (Tahir et al. 2013; Deng et al. 2019). We found that hybrid necrosis was highly dependent on light. Necrosis initiated in the leaf areas exposed to light, but not in the shaded leaf areas. It was reported that strong light exposure may promote the intracellular oxidative stress response, leading to increased production of reactive oxygen species and accelerated cell death (Apel and Hirt 2004). This may be one of the reasons for photo-induced hybrid necrosis.

Chlorophyll *b* plays an important role in light utilization under weak light conditions, light protection under strong light conditions, the assembly of chloroplast antenna complexes, the accumulation and assembly of pigment protein complexes in granular membranes, and the maintenance of the stability of light harvesting complex II (LHCII). Previous studies have shown that many angiosperm mutants with insufficient chlorophyll *b* biosynthesis exhibit increased photosensitivity. The *Chlb* deficiency mutant displayed a strong oxidative stress response under strong light (Voitsekhovskaja and Tyutereva 2015). STH59-2 and STH59-1 are two amphiploids from the same parent *H. villosa* accession CI084 due to the heterogeneity of *H. villosa*. STH59-2, with a relatively low chlorophyll *b* content, displayed necrosis, while STH59-1, with a relatively high chlorophyll b content, displayed no necrosis. It was speculated that STH59-2 exhibited greater photosensitivity than STH59-1, which was perhaps correlated with necrosis under strong light.

### Amphiploids derived from different *H. villosa* accessions are perfect materials for mapping trait-controlled genes

In the previous studies, the elite genes of *H. villosa* were often mapped by creating chromosome addition lines, substitution lines, translocation lines or introgression lines in the background of bread wheat to investigate the correlation between the introduced chromosome and phenotype occurring at the cytological level. In this way, multiple resistance-related and quality-related genes have been mapped. However, this method of gene discovery is time-consuming and not suitable for the large-scale exploration of *H. villosa* genes. In this study, we optimized a wide hybridization, protocol and performed large-scale hybridization between *T. durum* and 33 *H. villosa* accessions. The phenotypes of the F_1_ plants or the amphiploids derived from different *H. villosa* accessions, including those from the same *H. villosa* accession, showed a wide range of diversity. This observation highlights the high genetic diversity of different *H. villosa* accessions and the high heterogeneity of some *H. villosa* accessions.

The creation of homozygous amphiploids on a large scale makes it convenient to identify and map elite genes from *H. villosa* by developing segregating populations between different amphiploids. In addition to the diverse phenotypes of hybrid necrosis, the available amphiploids show wide variation in disease resistance, spike length, grain weight, plant height and protein content. Population construction using diverse amphiploids combined with the high-throughput development of molecular markers has made it quick to map elite genes from *H. villosa*. This approach facilitates the development of alien chromosome introgression lines carrying target genes.

### Supplementary Information

Below is the link to the electronic supplementary material.

Supplementary file1 (DOCX 17632 kb)
