# Supplementary material for: Phenotypic characterization and gene mapping of hybrid necrosis in Triticum durum–Haynaldia villosa amphiploids
Source: Theor Appl Genet. 2024 Jul 15;137(8):185. doi: 10.1007/s00122-024-04691-0 (PMC11249415; doi:10.1007/s00122-024-04691-0)
Supplement: Supplementary file 1 — Supplementary file1 (DOCX 17632 kb) [file 122_2024_4691_MOESM1_ESM.docx]

**Supporting information**

**
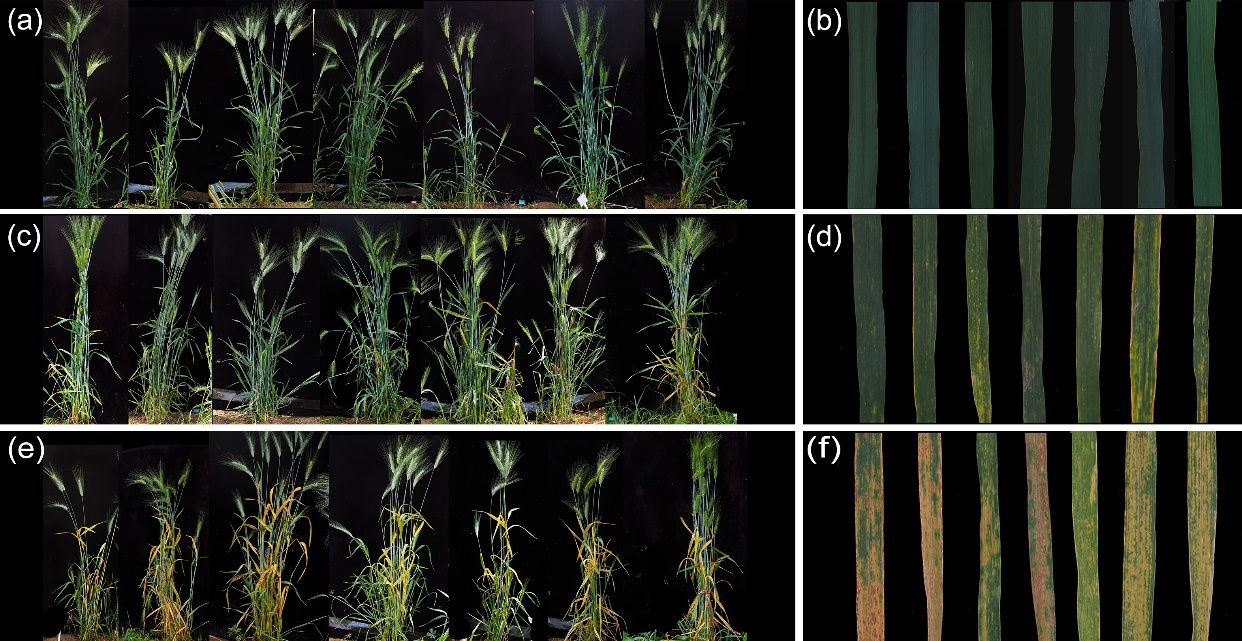
Fig. S1** **The whole plants and the appropriate leaves phenotypes of the F_1_ triploid (ABV) from durum wheat and *H. villosa* hybridization at the adult stage**

**a-b** The whole plants and leaves presented normal phenotypes. **c-d** The whole plants and leaves presented moderate necrotic phenotypes. **e-f** The whole plants and leaves presented severe necrotic phenotypes

**
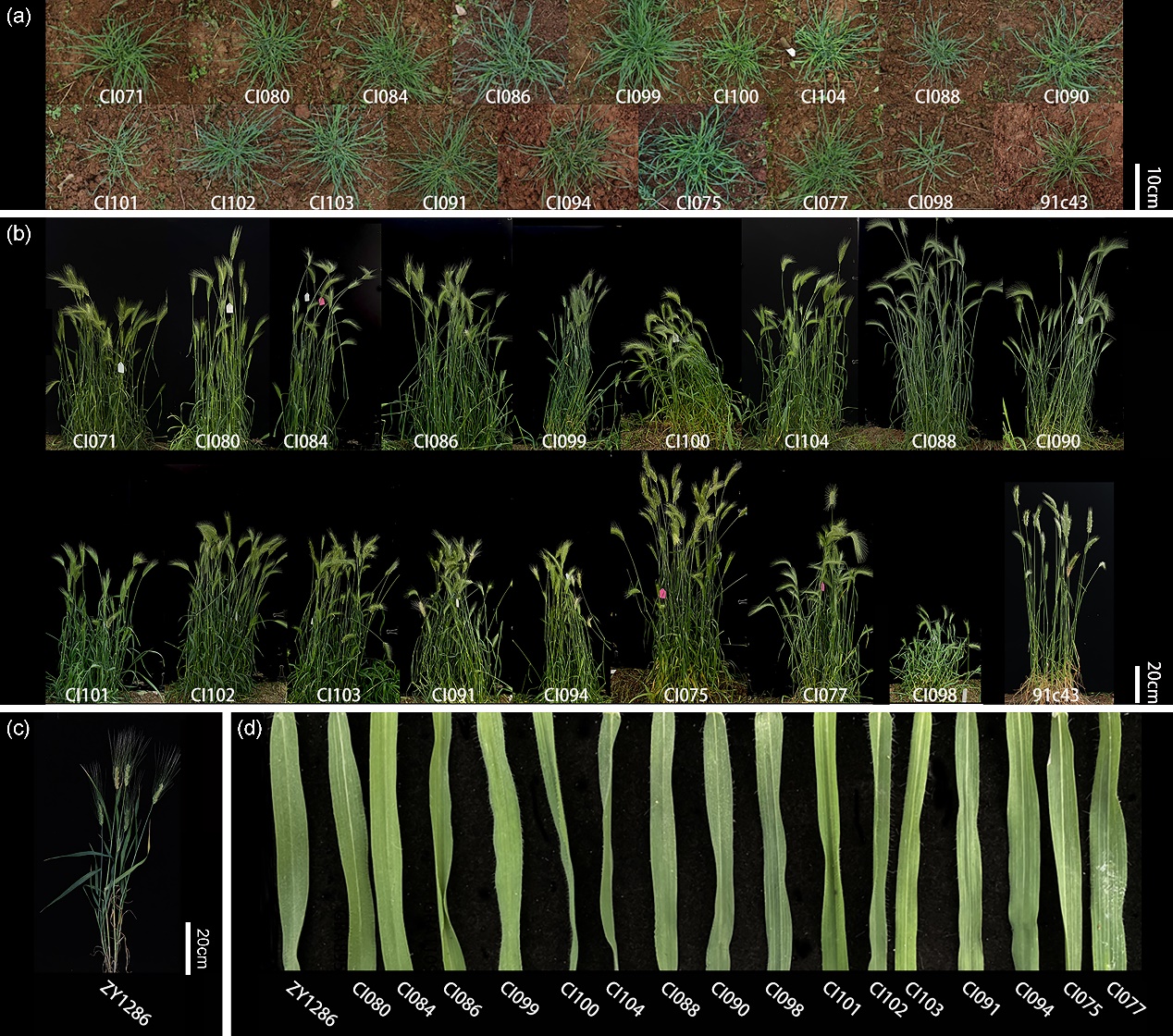
Fig. S2 Photos of the female parent *T. durum* *cv*. ZY1286 plants and different accession of the male parents *H. villosa* plants at the seedling stage and the adult stage**

**a** Seedling plants of different accessions of *H. villosa* (names start with CI). **b-c** Adult plants of different accessions of *H. villosa* and durum wheat ‘ZY1286’, respectively. **d** Leaves of durum wheat ‘ZY1286’ and different accessions of *H. villosa* at the adult stage

**Fig. S3 Development stages of necrosis initiation detected in amphiploids derived from different *H. villosa* accessions**


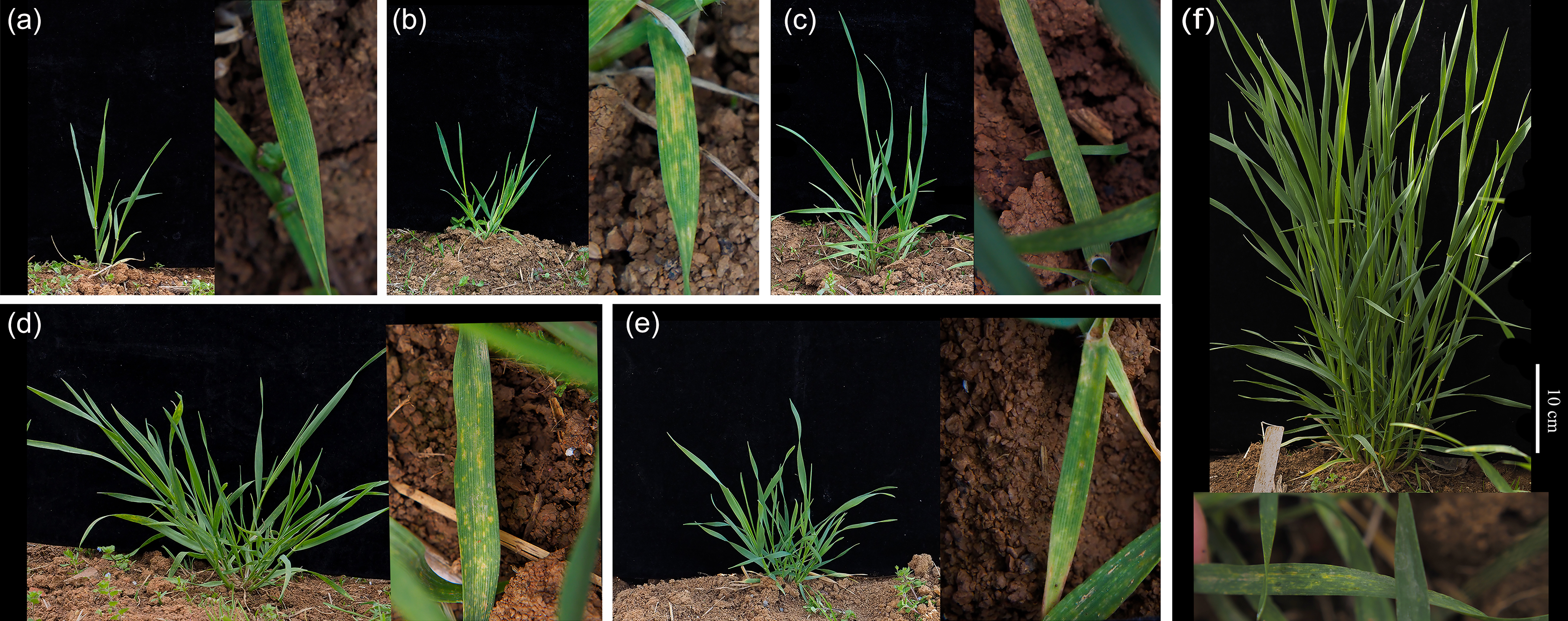


**a-b** Necrosis symptoms of amphiploid STH75-3 and STH73-3 at 2-3 tillers stage. **c-d** Necrosis symptoms of amphiploid STH77-4, STH79-2 and STH76-3 at 5-8 tillers stage. **f** Necrosis symptoms of amphiploid STH50-3 at booting stage

**Fig. S4 Expansion of necrosis with the advance of growth stage on amphiploid plants developed from different *H. villosa* accessions**


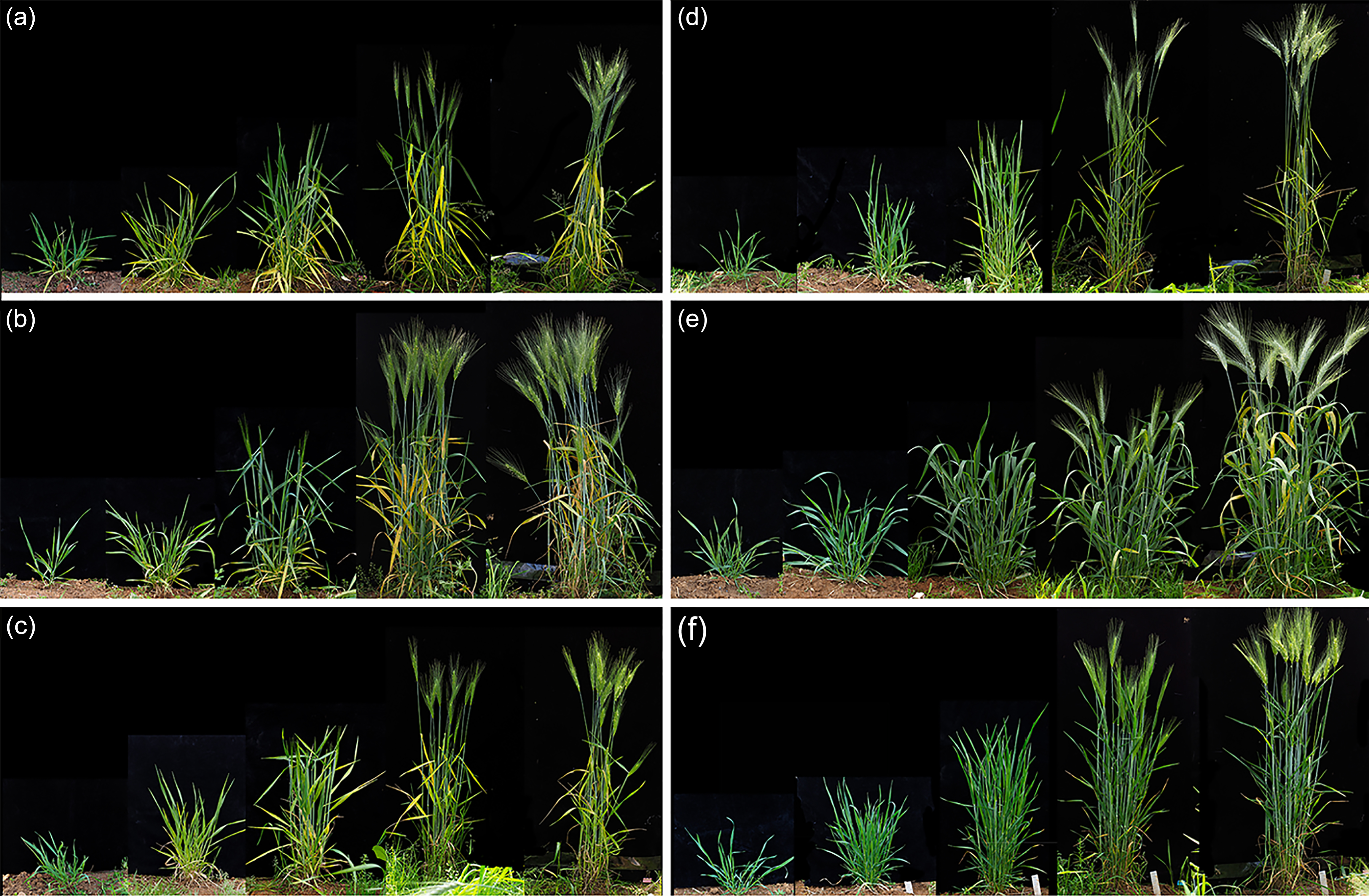


**a-f** Plant photos of the amphiploids STH75-3, STH73-3, STH77-4, STH79-2, STH76-3 and STH50-3, respectively. In each photo, from left to right represent the tillering stage, the jointing stage, the booting stage, the heading stage, and the filling stage

**Fig. S5 Leaves symptoms in the same leaf position of the amphiploids plants from different *H. villosa* accessions**


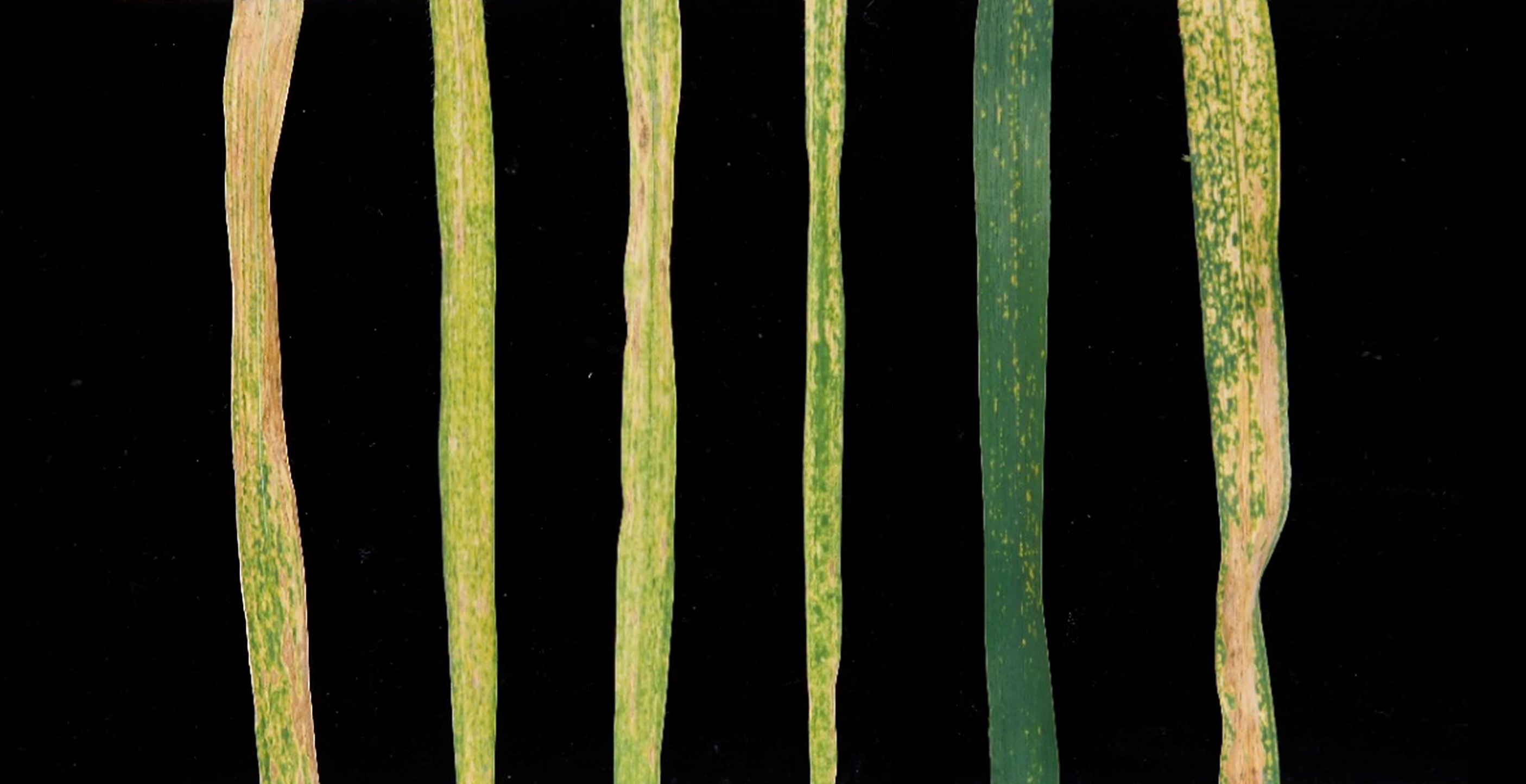


From left to right are the leaves of the amphiploids STH75-3, STH73-3, STH77-4, STH79-2, STH50-3, STH76-3

**Fig. S6 Leaf symptom comparison of STH59-1 and STH59-2 at seedling and tillering stage**


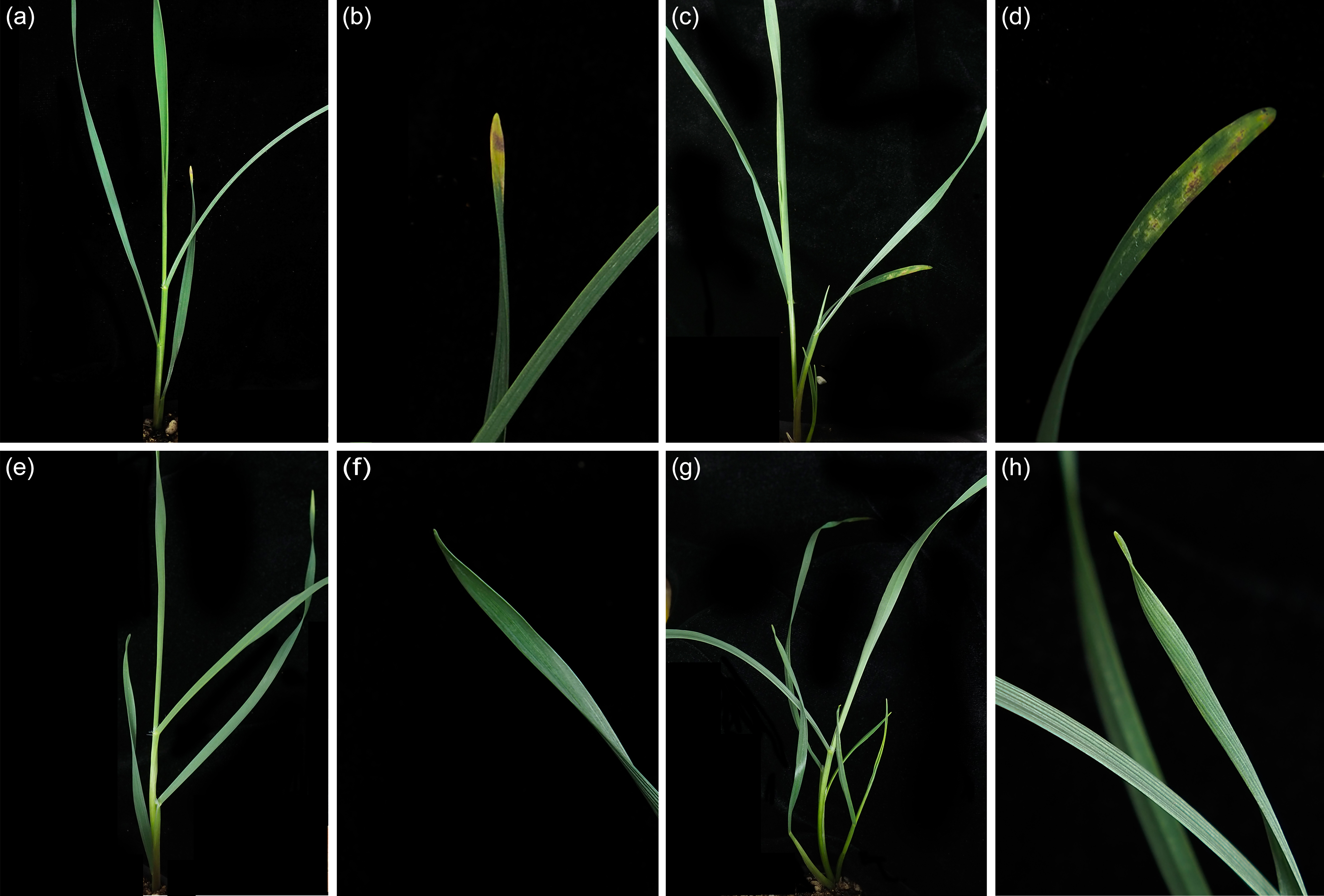


**a-b** Phenotype of leaf necrosis at four-leaf stage of STH59-2. **c-d** Phenotype of leaf necrosis at tillering stage of STH59-2. **e-f** Normal phenotype of leaves at four-leaf stage of STH59-1. **g-h** Normal phenotype of leaves at tillering stage of STH59-1

**
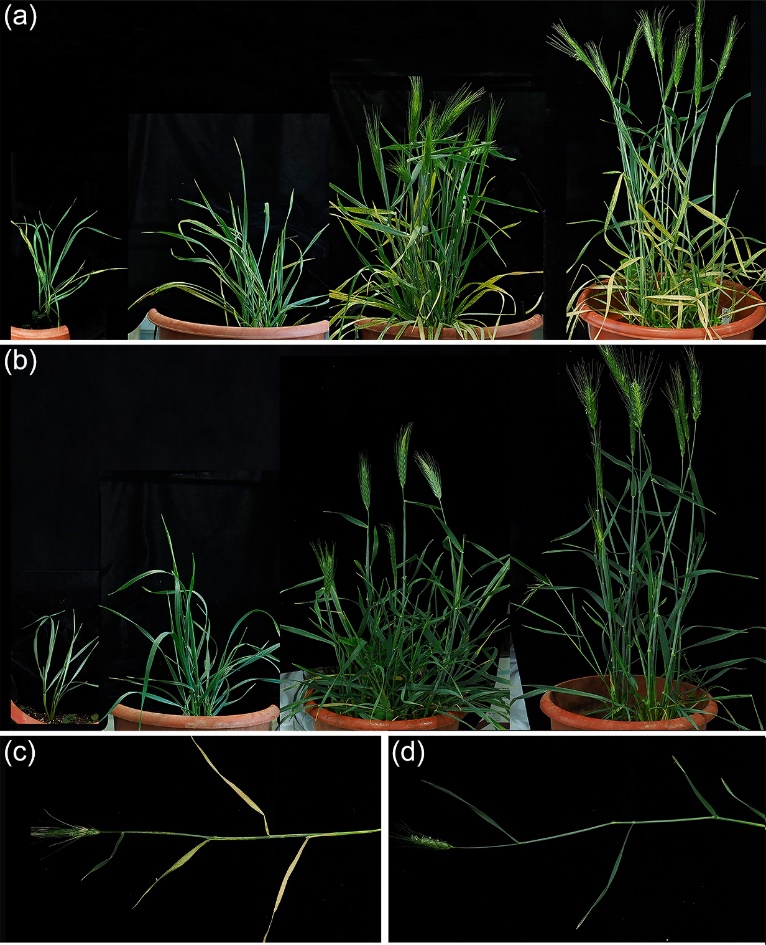
Fig. S7 Plant photos of the amphiploid plants of STH59-1 and STH59-2 with the advance of growth stage**

**a** STH59-2 plants with leaf necrosis at tiller stage, jointing stage, heading stage, and maturity stage. **b** STH59-1 plants with normal leaves at tiller stage, jointing stage, heading stage, and maturity stage. **c** One tiller separated from STH59-2 plant with leaf necrosis. **d** One tiller separated from STH59-1 plant with normal leaves

**
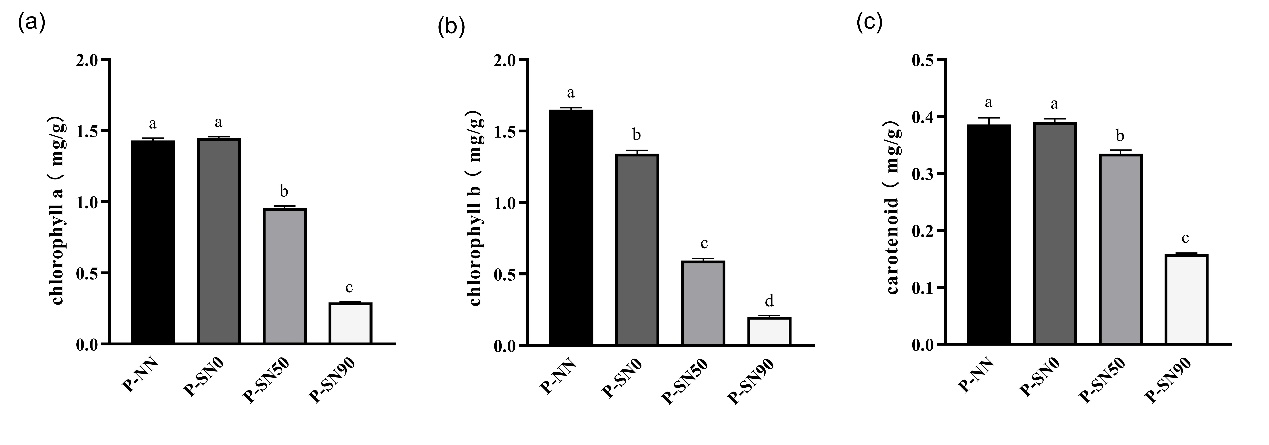
Fig. S8 Comparison of** **chlorophyll content between leaves of necrotic STH59-2 and normal STH59-1**

**a** chlorophyll a. **b** chlorophyll b. **c** carotenoid. P-NN represents normal leaves of STH59-1, P-SN0, P-SN50 and P-SN90 represent normal leaves, 50%-necrotic leaves and 90%- necrotic leaves of STH59-2. Letters indicate significant differences determined using Student’s t-test (P < 0.05). Error bars represent the SD

**Fig. S9 Leaf necrosis of individuals from F2:3 population derived from STH59-1 and STH59-2 hybridization**


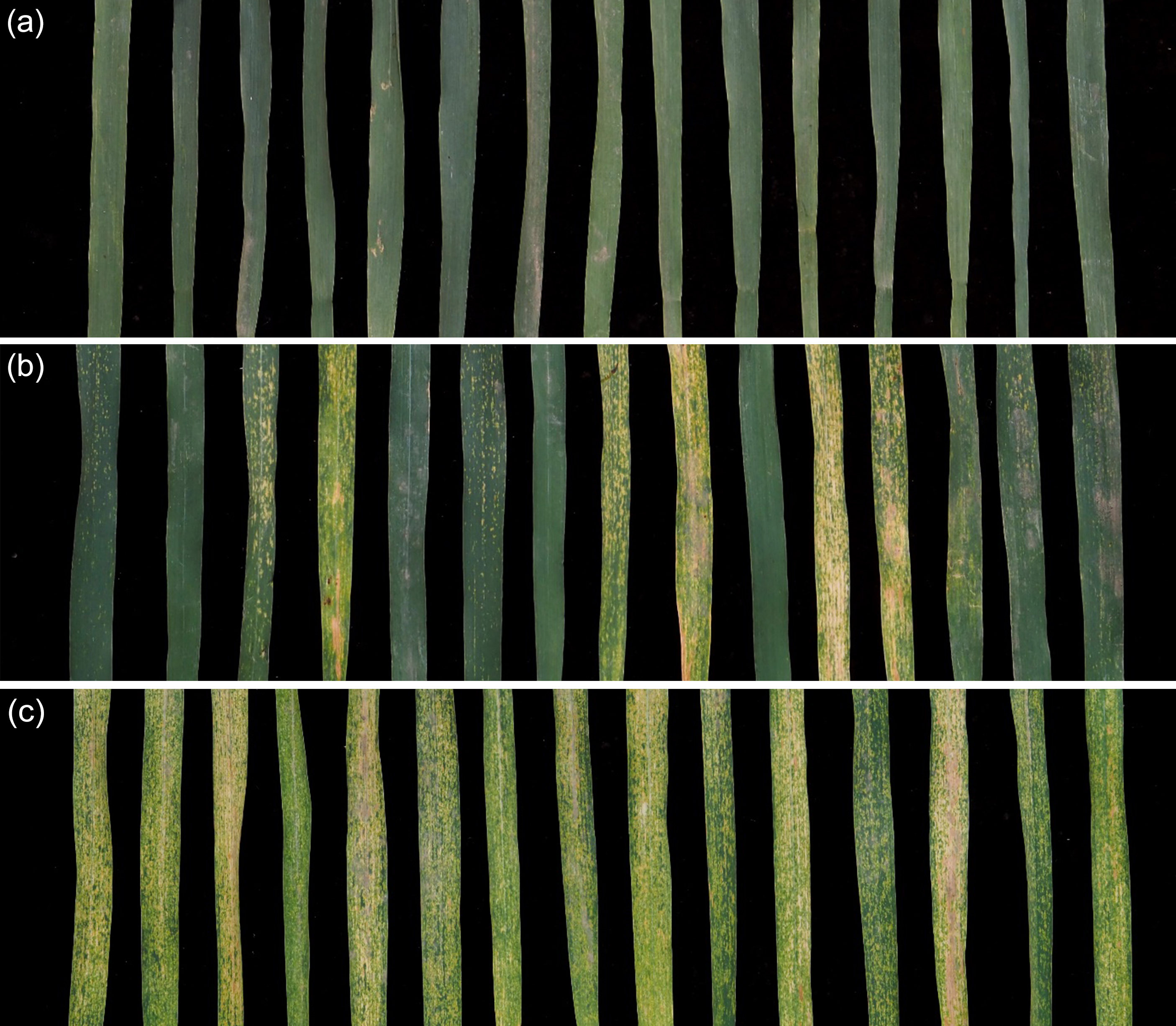


**a** Leaf phenotype of F_2:3_ derived from normal F_2_ plant. **b** Leaf phenotype of F_2:3_ derived from slightly necrotic F_2_ plant. **c** Leaf phenotype of F_2:3_ derived from severely necrotic F_2_ plant

**
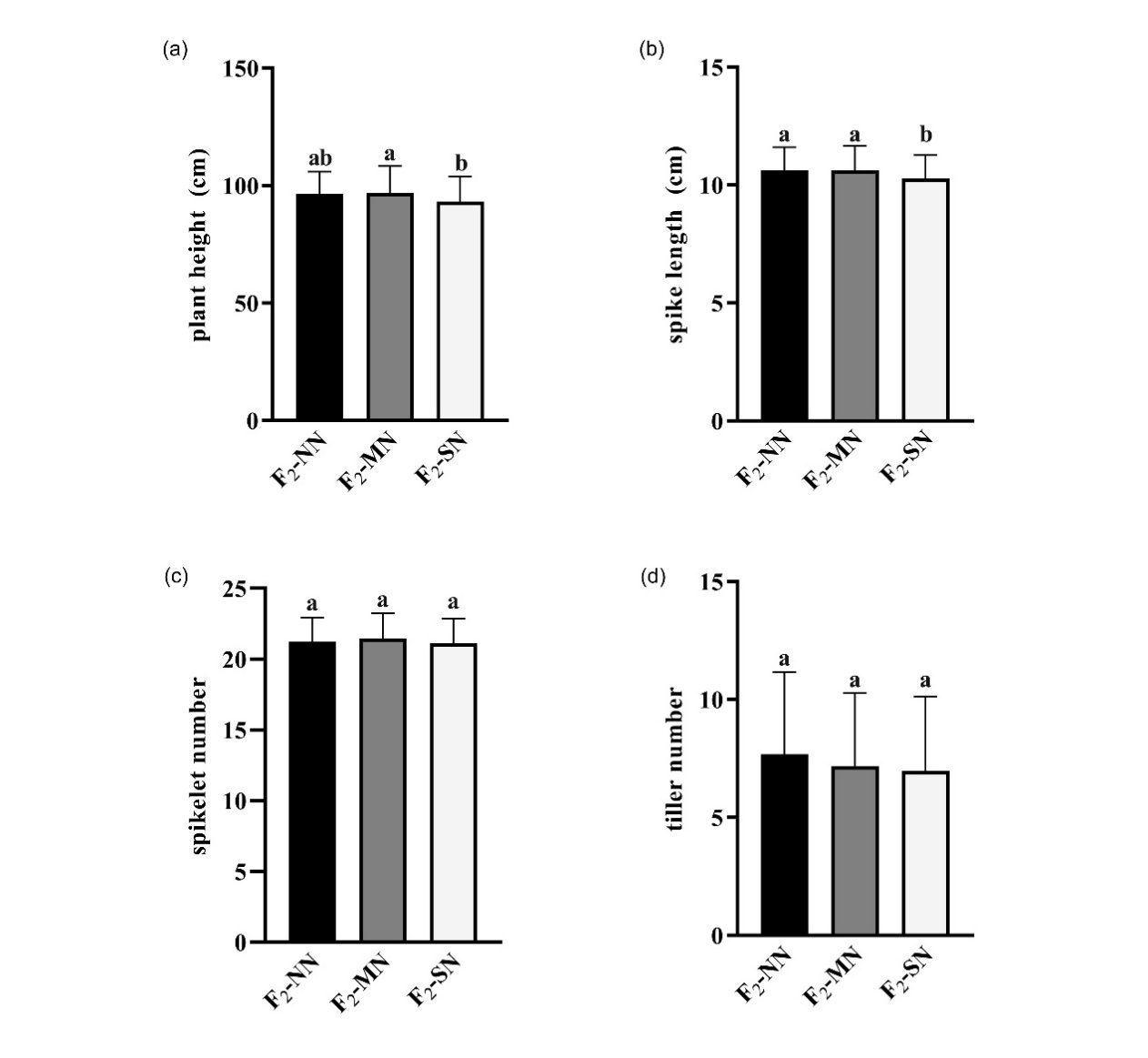
Fig. S10 Comparison of agronomic traits between F_2_ individuals with different necrotic degrees**

**a** Plant height. **b** Spike length. **c** Spike number. **d** Tiller number. F_2_- NN, F_2_-MN and F_2_-SN represent non-necrotic, moderate necrotic and severe necrotic, respectively. Letters indicate significant differences determined using Student’s t-test (P < 0.05). Error bars represent the SD

**Fig. S11 Comparison of grain characters between F_2_ individuals with different necrosis degrees**


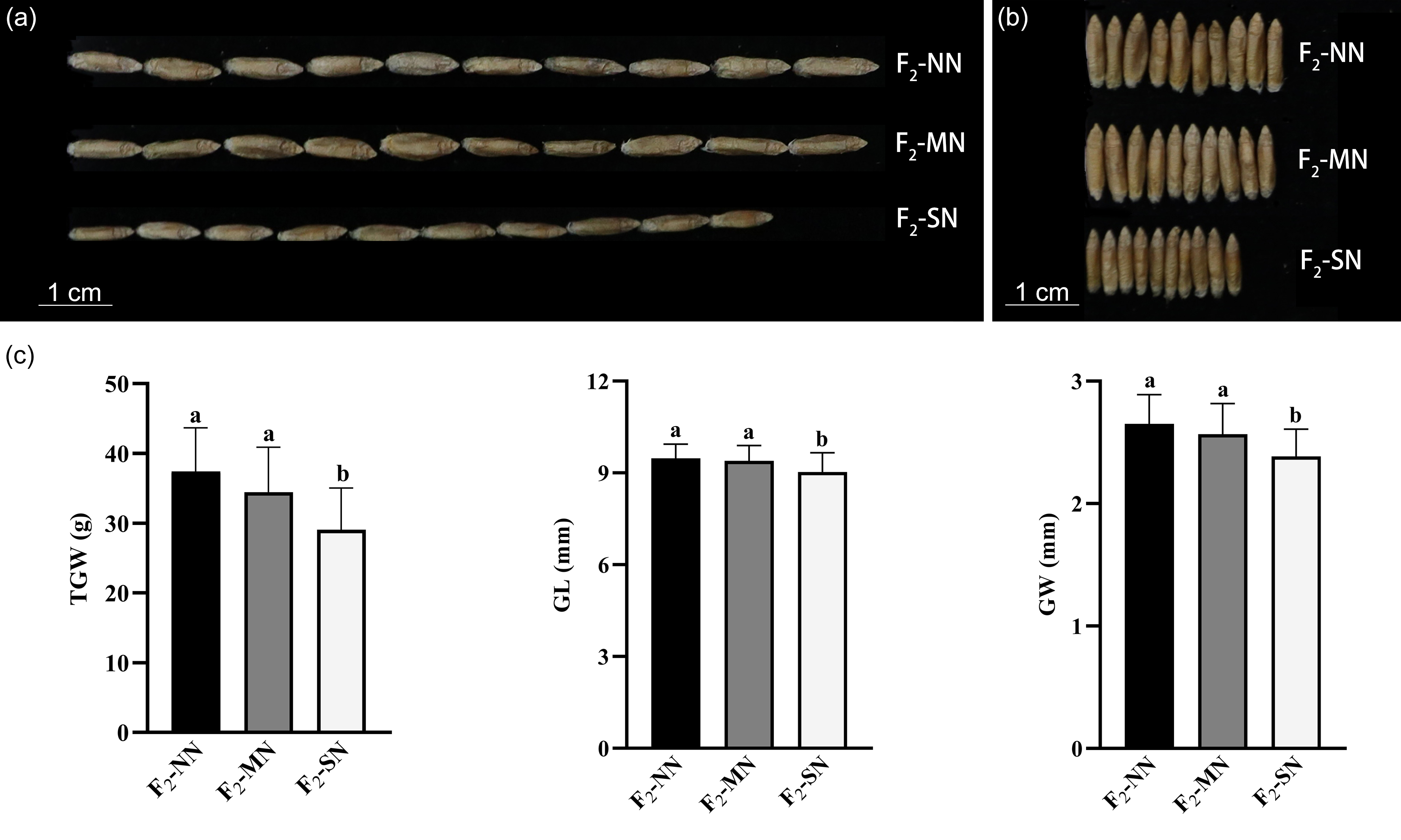


**a-b** Grain length and grain width comparison. Scale bar= 1cm. **c** Statistical analysis of thousand grain weight (TGW), grain length (GL) and grain width (GW). F_2_- NN, F_2_-MN and F_2_-SN represent non-necrotic, moderate necrotic and severe necrotic, respectively. Letters indicate significant differences determined using Student’s t-test (P < 0.05). Error bars represent the SD

**
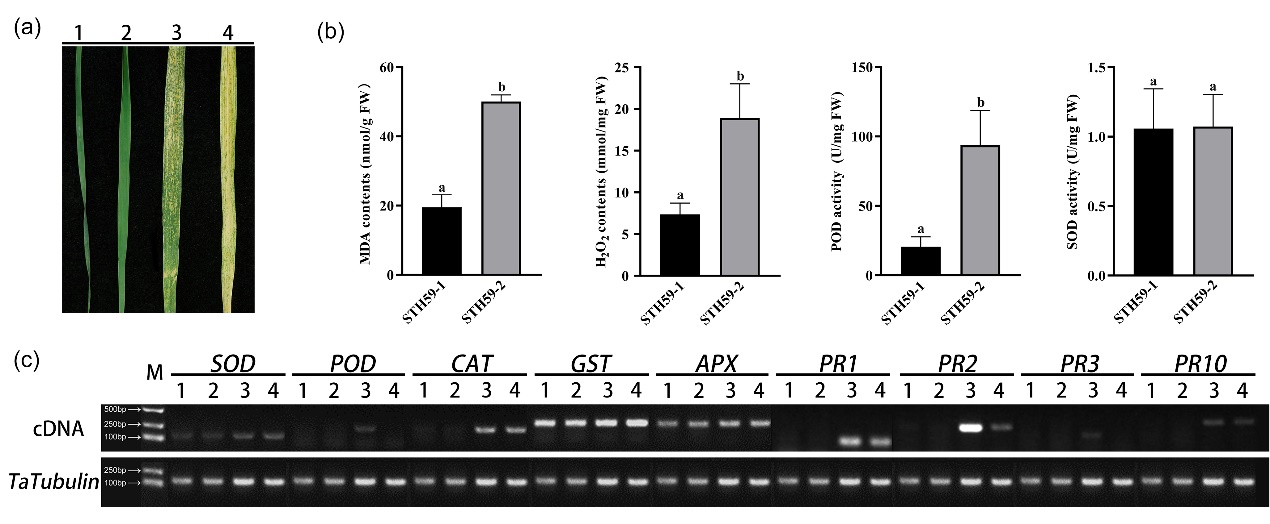
****Fig. S12 Determination of oxidation and antioxidant indexes and expression of defense and antioxidant related genes in different types of leaves from STH59-1and STH59-2**

**a** 1 and 2 represent normal leaves of STH59-1 and STH59-2, respectively. 3 and 4 represent 50%-necrotic and 90%- necrotic leaves of STH59-2, respectively. The four types of leaves were sampled for RT-PCR analysis. **b** MDA and H_2_O_2_, contents in necrotic leaves from STH59-2 and the normal leaves of STH59-1 were tested. Enzyme activity of POD and SOD were measured in necrotic leaves from STH59-2 and the normal leaves of STH59-1. Letters indicate significant differences determined using Student’s t-test (P < 0.05). Error bars represent the SD. **c** Expression pattern of *SOD*, *POD*, *CAT*, *GST*, *APX*, *PR1*, *PR2*, *PR3* and *PR10* in the four types of leaves described in **a**


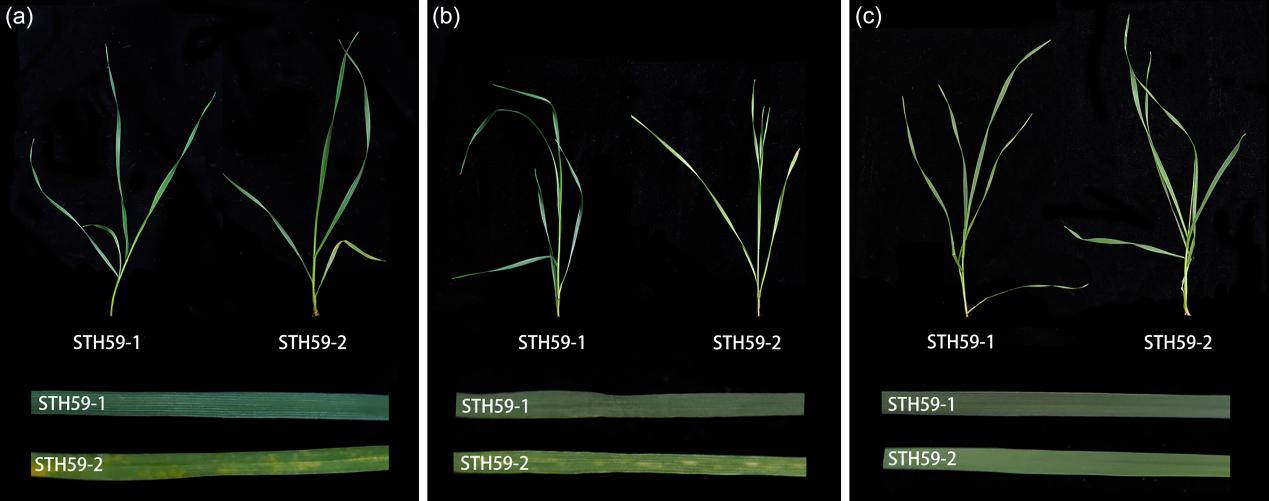
**Fig. S13 Phenotypic identification of leaf necrosis at seedling stage under different growing temperatures**

**a-c** Four-leaf stage seedling and leaf phenotypes of STH59-1 and STH59-2. Necrosis of STH59-2 was observed earlier when grew at 14 ℃ (**a**) than that grew at 23 ℃ (**b**), but it was delayed when grew at 30 ℃ (**c**), indicating that lower temperature could accelerate leaf necrosis, while higher temperature could delay leaf necrosis


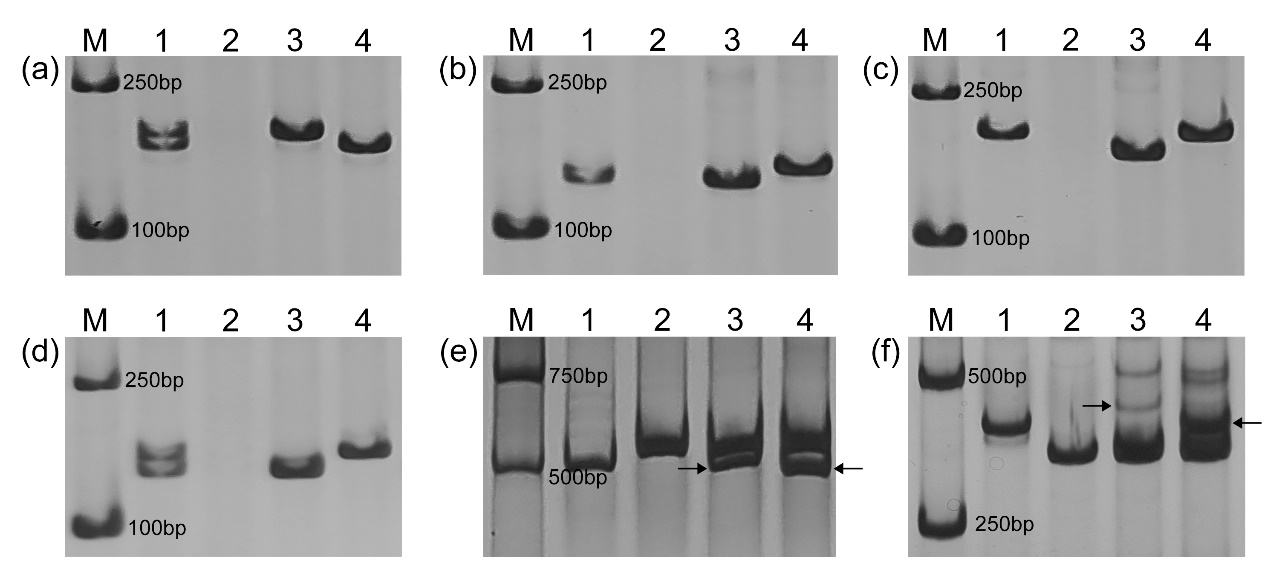


**Fig. S14 The 6 polymorphic markers tested in the CI084, ZY1286, STH59-1 and STH59-2**

M, 1, 2, 3 and 4 represent DNA Marker DL2000 (TAKARA, Japan), CI084, ZY1286, STH59-1 and STH59-2, respectively. In CI084, the markers *InDel4* (**a**) and *InDel9* (**d**) show heterozygous in the tested locus, while the markers *InDel6* (**b**), *InDel8* (**c**), *2EST-895* (**e**) and *SNP-605* (**f**) show homozygous in the tested locus. The arrows indicated the polymorphism bands

**

Fig. S15 The co-segregation marker *5B-InDel385* of *Ne1* was** **detected in ZY1286**

M, 1, 2 and 3 represent DNA Marker DL2000 (TAKARA, Japan), ZY1286, Chinese Spring and Zhoumai 18, respectively. The specific band of *Ne1* was amplified from ZY1286 and the positive control, wheat *cv.* Chinese Spring, while was not amplified from the negative control, wheat *cv.* Zhoumai 18

**Table S1 Investigation of hybrid necrosis of durum wheat-*H. villosa* F_1_ triploids and corresponding amphiploids**

| Accessions of the F_1_ plants |  | Ratio of chlorosis area of the top second leaf (%) in F_1_ plants | Ratio of chlorosis area of top second leaf (%) in corresponding amphiploids | Accessions of corresponding *H. villosa* |
| --- | --- | --- | --- | --- |
| 22SH1-1 |  | 93.12 ± 4.1 | 63.65 ± 0.18 | CI074 |
| 22SH1-2 |  | 96.84 ± 2.26 | - |  |
| 22SH1-3 |  | 90.55 ± 1.89 | - |  |
| 22SH2-1 |  | 0.00 | 0.00 | CI076 |
| 22SH2-2 |  | 0.00 | 0.00 |  |
| 22SH2-3 |  | 0.00 | - |  |
| 22SH2-4 |  | 86.36 ± 7.32 | - |  |
| 22SH2-5 |  | 85.92 ± 5.31 | 80.18 ± 0.62 |  |
| 22SH2-6 |  | 91.65 ± 3.44 | - |  |
| 22SH2-7 |  | 0.00 | - |  |
| 22SH2-8 |  | 0.00 | - |  |
| 22SH3-1 |  | 89.89 ± 4.36 | 60.56 ± 3.21 | CI079 |
| 22SH3-2 |  | 89.53 ± 5.41 | 88.42 ± 2.18 |  |
| 22SH3-3 |  | 97.96 ± 0.7 | - |  |
| 22SH3-4 |  | 0.00 | 0.00 |  |
| 22SH3-5 |  | 93.14 ± 1.76 | - |  |
| 22SH3-6 |  | 100.00 | - |  |
| 22SH4-1 |  | 0.00 | - | CI085 |
| 22SH4-2 |  | 0.00 | 0.00 |  |
| 22SH4-3 |  | 0.00 | 0.00 |  |
| 22SH5-1 |  | 0.00 | - | CI087 |
| 22SH5-2 |  | 80.94 ± 2.53 | - |  |
| 22SH5-3 |  | 100.00 | - |  |
| 22SH5-4 |  | 0.00 | 0.00 |  |
| 22SH5-5 |  | 77.08 ± 2.96 | - |  |
| 22SH5-6 |  | 77.93 ± 3.28 | 80.35 ± 1.44 |  |
| 22SH5-7 |  | 100.00 | - |  |
| 22SH5-8 |  | 76.66 ± 2.71 | - |  |
| 22SH5-9 |  | 83.92 ± 3.00 | - |  |
| 22SH5-10 |  | 76.30 ± 1.61 | - |  |
| 22SH6-1 |  | 100.00 | - | CI092 |
| 22SH6-2 |  | 75.92 ± 4.21 | - |  |
| 22SH6-3 |  | 75.63 ± 3.57 | - |  |
| 22SH6-4 |  | 0.00 | - |  |
| 22SH6-5 |  | 67.53 ± 2.85 | - |  |
| 22SH6-6 |  | 0.00 | - |  |
| 22SH7-1 |  | 0.00 | - | CI093 |
| 22SH7-2 |  | 15.89 ± 1.37 | 21.75 ± 3.22 |  |
| 22SH7-3 |  | 9.74 ± 2.18 | 36.45 ± 4.70 |  |
| 22SH7-4 |  | 19.69 ± 4.05 | 13.22 ± 2.98 |  |
| 22SH7-5 |  | 0.00 | - |  |
| 22SH7-6 |  | 21.08 ± 5.94 | 10.18 ± 0.82 |  |
| 22SH7-7 |  | 14.00 ± 1.36 | 3.98 ± 0.34 |  |
| 22SH7-8 |  | 13.47 ± 4.55 | 26.88 ± 3.12 |  |
| 22SH8-1 |  | 0.00 | - | CI096 |
| 22SH8-2 |  | 89.15 ± 4.46 | 82.58 ± 3.45 |  |
| 22SH8-3 |  | 62.06 ± 5.84 | - |  |
| 22SH8-4 |  | 0.00 | 0.00 |  |
| 22SH8-5 |  | 0.00 | - |  |
| 22SH8-6 |  | 0.00 | 0.00 |  |
| 22SH8-7 |  | 48.21 ± 5.37 | - |  |
| 22SH9-1 |  | 13.67 ± 4.77 | - | CI097 |
| 22SH9-2 |  | 15.02 ± 5.31 | - |  |
| 22SH9-3 |  | 63.68 ± 6.27 | 72.68 ± 1.24 |  |
| 22SH9-4 |  | 12.06 ± 2.03 | 15.33 ± 0.25 |  |
| 22SH9-5 |  | 72.66 ± 4.90 | - |  |
| 22SH9-6 |  | 3.70 ± 0.86 | - |  |
| 22SH10-1 |  | 5.27 ± 1.77 | 22.54 ± 2.36 | CI106 |
| 22SH10-2 |  | 16.96 ± 2.55 | - |  |
| 22SH10-3 |  | 31.70 ± 1.21 | - |  |
| 22SH10-4 |  | 0.00 | - |  |
| 22SH10-5 |  | 13.70 ± 3.61 | 26.42 ± 3.22 |  |
| 22SH10-6 |  | 3.49 ± 0.72 | - |  |
| 22SH11-1 |  | 75.76 ± 4.66 | 64.82 ± 1.62 | CI108 |
| 22SH11-2 |  | 68.75 ± 8.85 | - |  |
| 22SH11-3 |  | 84.67 ± 5.29 | 85.44 ± 0.57 |  |
| 22SH11-4 |  | 85.27 ± 5.05 | 89.43 ± 3.54 |  |
| 22SH11-5 |  | 0.00 | - |  |
| 22SH11-6 |  | 84.81 ± 4.95 | - |  |
| 22SH11-7 |  | 0.00 | - |  |
| 22SH12-1 |  | 39.68 ± 6.61 | - | CI109 |
| 22SH12-2 |  | 100.00 | - |  |
| 22SH12-3 |  | 73.61 ± 1.56 | - |  |
| 22SH12-4 |  | 20.19 ± 1.14 | - |  |
| 22SH12-5 |  | 100.00 | - |  |
| 22SH12-6 |  | 46.95 ± 6.99 | - |  |
| 22SH13-1 |  | 48.06 ± 6.92 | - | CI111 |
| 22SH13-2 |  | 0.00 | 0.00 |  |
| 22SH13-3 |  | 54.24 ± 5.08 | 65.75 ± 1.89 |  |
| 22SH13-4 |  | 61.43 ± 5.24 | 46.88 ± 0.69 |  |
| 22SH13-5 |  | 100.00 | - |  |
| 22SH13-6 |  | 0.00 | - |  |
| 22SH13-7 |  | 0.00 | - |  |

**Table S2 Investigation of hybrid necrosis of durum wheat-*H. villosa* amphiploids**

| Accessions of amphiploids | Ratio of chlorosis area of the top second leaf (%) | Accessions of corresponding *H. villosa* |
| --- | --- | --- |
| STH29-1 | 0.00 | CI054 |
| STH29-2 | 0.00 |  |
| STH33-1 | 0.00 | CI058 |
| STH36-1 | 0.00 | CI061 |
| STH36-2 | 18.32 ± 4.92 |  |
| STH36-3 | 0.00 |  |
| STH37-1 | 0.00 | CI062 |
| STH55-1 | 0.00 | CI080 |
| STH55-2 | 0.00 |  |
| STH55-3 | 18.39 ± 8.24 |  |
| STH55-4 | 7.30 ± 0.18 |  |
| STH55-5 | 13.36 ± 5.43 |  |
| STH55-6 | 11.31 ± 2.04 |  |
| STH50-1 | 0.00 | CI075 |
| STH50-2 | 3.7 ± 2.57 |  |
| STH50-3 | 8.04 ± 1.44 |  |
| STH50-4 | 0.00 |  |
| STH50-5 | 0.00 |  |
| STH52-1 | 0.00 | CI077 |
| STH52-2 | 0.00 |  |
| STH52-3 | 0.00 |  |
| STH52-4 | 0.00 |  |
| STH52-5 | 18.71 ± 6.16 |  |
| STH66-1 | 30.72 ± 7.17 | CI091 |
| STH66-2 | 0.00 |  |
| STH66-3 | 21.16 ± 3.52 |  |
| STH66-4 | 11.97 ± 8.30 |  |
| STH66-5 | 17.82 ± 2.47 |  |
| STH61-1 | 9.45 ± 3.89 | CI086 |
| STH61-2 | 0.00 |  |
| STH61-3 | 0.00 |  |
| STH74-1 | 89.40 ± 2.19 | CI099 |
| STH74-2 | 68.39 ± 5.49 |  |
| STH74-3 | 0.00 |  |
| STH74-4 | 58.44 ± 7.59 |  |
| STH74-5 | 27.25 ± 1.91 |  |
| STH75-1 | 41.70 ± 1.35 | CI100 |
| STH75-2 | 81.43 ± 3.31 |  |
| STH75-3 | 74.52 ± 9.20 |  |
| STH75-4 | 0.00 |  |
| STH46-1 | 0.00 | CI071 |
| STH46-2 | 25.52 ± 5.67 |  |
| STH46-3 | 0.00 |  |
| STH46-4 | 0.00 |  |
| STH46-5 | 0.00 |  |
| STH79-1 | 78.47 ± 3.50 | CI104 |
| STH79-2 | 79.38 ± 6.03 |  |
| STH79-3 | 0.00 |  |
| STH79-4 | 63.51 ± 1.23 |  |
| STH79-5 | 0.00 |  |
| STH65-1 | 14.21 ± 2.23 | CI090 |
| STH65-2 | 9.15 ± 5.71 |  |
| STH65-3 | 0.00 |  |
| STH65-4 | 12.11 ± 2.20 |  |
| STH65-5 | 15.30 ± 1.68 |  |
| STH65-6 | 0.00 |  |
| STH69-1 | 14.87 ± 4.07 | CI094 |
| STH77-1 | 87.23 ± 2.02 | CI102 |
| STH77-2 | 82.53 ± 5.14 |  |
| STH77-3 | 79.23 ± 2.01 |  |
| STH77-4 | 61.67 ± 8.14 |  |
| STH77-5 | 72.54 ± 5.25 |  |
| STH77-6 | 0.00 |  |
| STH63-1 | 19.74 ± 8.83 | CI088 |
| STH78-1 | 68.17 ± 5.46 | CI103 |
| STH78-2 | 10.79 ± 0.61 |  |
| STH78-3 | 21.98 ± 4.71 |  |
| STH73-1 | 56.34 ± 11.59 | CI098 |
| STH73-2 | 17.86 ± 8.93 |  |
| STH73-3 | 74.28 ± 3.78 |  |
| STH73-4 | 71.98 ± 4.89 |  |
| STH73-5 | 66.23 ± 9.18 |  |
| STH76-1 | 55.58 ± 6.78 | CI101 |
| STH76-2 | 15.71 ± 1.24 |  |
| STH76-3 | 81.98 ± 5.44 |  |
| STH59-1 | 0.00 | CI084 |
| STH59-2 | 91.40 ± 1.19 |  |
| AABBVV | 20.32 ± 1.10 | 91c43 |

**Table S3 Comparison of agronomic traits between synthetic amphiploid and their parents**

| Materials | Plant Height (cm) | Effective Tillers | Spike Length  (cm) | Spikelet Number |
| --- | --- | --- | --- | --- |
| ZY1286 | 74.7±9.0 a | 8.8±2.2 a | 8.8±0.5 a | 20.4±1.1 a |
| CI084 | 111.9±1.6 c | 32.0±7.5 b | 10.1±0.2 b | 25.3±0.9 b |
| STH59-1 | 90.8±7.7 b | 9.6±3.9 a | 10.8±0.6 c | 21.3±1.3 ab |
| STH59-2 | 97.5±4.5 b | 14.2±3.3 a | 10.7±0.7 c | 23.4±1.5 b |

**Table S4 Statistics of exome capture sequencing**

| Sample | Raw_bases | Clean_bases | Raw_reads | Clean_reads | Reads_mapped | Align_rate (%) | |
| --- | --- | --- | --- | --- | --- | --- | --- |
| necrosis-pool | 14,478,837,600 | 13,105,812,158 | 96,525,584 | 96,497,004 | 93,099,316 | | 96.48 |
| no necrosis-pool | 13,940,317,500 | 12,706,922,412 | 92,935,450 | 92,910,042 | 89,840,580 | | 96.70 |

**Table S5 The information of the predicted interval**

| chrom | start | end | length | nSNPs | avgSNPs_Mb |
| --- | --- | --- | --- | --- | --- |
| chr2V | 535333959 | 606336595 | 71002636 | 14012 | 197 |

**Table S6 The sequences of primers used in this study**

| Primer name | Sequence (5'→3') |
| --- | --- |
| InDel4-F | CACGGCCTCAAGAATAGAGC |
| InDel4-R | GGGCTTGAGGAGTGTTTTGG |
| InDel6-F | AGTTTCTACTCCTGCCGATC |
| InDel6-R | CCACCACCAAATTCGCTAGA |
| InDel8-F | CAGAGCGGTTCAGGTTCAG |
| InDel8-R | GGAGAGGAGTTACATGGTGG |
| InDel9-F | AGGCGAAGATTCAACGAAAGA |
| InDel9-R | CTCAACTCAAACCTCCTCCG |
| 2EST-837-F | CCATGGGGTACGTGAAGAAC |
| 2EST-837-R | AAGCTCGTCCGTGGTATGTC |
| 2EST-895-F | GCATCTGCAGGGACCATAAT |
| 2EST-895-R | ATAGGAAGCCAGCCCAGAAT |
| SNP605-in-F | ACAAAATTTCATCAAGCTAACAGAACAA |
| SNP605-in-R | AGTTTTCCACCTTAATTCCGCC |
| SNP605-out-F | GCAAAGTCAATCAACTCACCTCC |
| SNP605-out-R | ACGTGTCTTCTGCATCAAGAGCT |
| CAT-F | TGCCTGTGTTTTTTATCCGAGA |
| CAT-R | CTGCTGATTAAGGTGTAGGTGTT |
| APX-F | GGTTTGAGTGACCAGGACATTG |
| APX-R | GCATCCTCATCCGCAGCAT |
| GST-F | GGAGCACAAGAGCCCCGAGC |
| GST-R | CGGGTTGTAGGTGTGCGCGT |
| SOD-F | AGAACCTCAAGCCCATCAGCG |
| SOD-R | CACCCATCCAGATCCTTGTAAAGC |
| POD-F | GCCCGTGACTCCGTTGTAGC |
| POD-R | GATCCTGTCCTTAAAGGTCCCACA |
| PR1-F | CTGGAGCACGAAGCTGCAG |
| PR1-R | CGAGTGCTGGAGCTTGCAGT |
| PR2-F | GCAGCTCTACAGGTCCAAGG |
| PR2-R | CGGCGATGTACTTGATGTTG |
| PR5-F | CAAGCAGTGGTATCAACGCAGAG |
| PR5-R | GTGAAGCCACAGTTGTTCTTGAT |
| PR10-F | ACGGAGCGGATGTGGAAG |
| PR10-R | GCCACCTGCGACTTGAGC |
| TaTubulin-F | GATGCAGCCAACAACTTCGCC |
| TaTubulin-R | CAGTTCCACCTCCAACAGCGT |
